# The response of mesophyll conductance to short-term variation in CO_2_ in the C_4_ plants *Setaria viridis* and *Zea mays*

**DOI:** 10.1093/jxb/erx464

**Published:** 2018-02-21

**Authors:** Nerea Ubierna, Anthony Gandin, Asaph B Cousins

**Affiliations:** School of Biological Sciences, Molecular Plant Sciences, Washington State University, Pullman, Washington, USA

**Keywords:** *A*-*C*_i_ curves, carbonic anhydrase, CO_2_, C_4_ photosynthesis, diffusional limitations, *in-vitro V*_pmax_, leakiness, mesophyll conductance, *Setaria viridis*, *Zea mays*

## Abstract

Mesophyll conductance (*g*_m_) limits rates of C_3_ photosynthesis but little is known about its role in C_4_ photosynthesis. If *g*_m_ were to limit C_4_ photosynthesis, it would likely be at low CO_2_ concentrations (*p*CO_2_). However, data on C_4_-*g*_m_ across ranges of *p*CO_2_ are scarce. We describe the response of C_4_-*g*_m_ to short-term variation in *p*CO_2_, at three temperatures in *Setaria viridis*, and at 25 °C in *Zea mays*. Additionally, we quantified the effect of finite *g*_m_ calculations of leakiness (ϕ) and the potential limitations to photosynthesis imposed by stomata, mesophyll, and carbonic anhydrase (CA) across *p*CO_2_. In both species, *g*_m_ increased with decreasing *p*CO_2_. Including a finite *g*_m_ resulted in either no change or increased ϕ compared with values calculated with infinite *g*_m_ depending on whether the observed ^13^C discrimination was high (*Setaria*) or low (*Zea*). Post-transitional regulation of the maximal PEP carboxylation rate and PEP regeneration limitation could influence estimates of *g*_m_ and ϕ. At *p*CO_2_ below ambient, the photosynthetic rate was limited by CO_2_ availability. In this case, the limitation imposed by the mesophyll was similar or slightly lower than stomata limitation. At very low *p*CO_2_, CA further constrained photosynthesis. High *g*_m_ could increase CO_2_ assimilation at low *p*CO_2_ and improve photosynthetic efficiency under situations when CO_2_ is limited, such as drought.

## Teaser

In two C_4_ species, mesophyll conductance increases with short-term exposure to decreasing *p*CO_2_ and limits photosynthetic capacity below ambient levels, whilst carbonic anhydrase imposes a further limitation only at very low *p*CO_2_.

## Introduction

In C_4_ plants photorespiration is reduced by concentrating CO_2_ around Rubisco (ribulose 1,5-bisphosphate carboxylase/oxygenase) (Edwards and Walker, 1983; Hatch, 1987; Sage, 2004). In Kranz-type C_4_ plants this is achieved with a compartmentalized two-carboxylation process: (1) in the cytosol of mesophyll cells, bicarbonate (HCO_3_^–^) and phospho*enol*pyruvate are fixed into four-carbon acids by phospho*enol*pyruvate carboxylase (PEPC) (Hatch *et al.*, 1967); and (2) in chloroplasts of the bundle-sheath cells the concentrated CO_2_ released from the decarboxylation of these acids is fixed by Rubisco.

Mesophyll conductance (*g*_m_) describes the movement of CO_2_ from stomata across the intercellular spaces to the sites of first carboxylation, which are the chloroplast stroma or mesophyll cytosol in C_3_ and C_4_ species, respectively (Evans and von Caemmerer, 1996). There is extensive research describing *g*_m_ in C_3_ species; however, C_4_-*g*_m_ is poorly understood because it is difficult to estimate. Traditionally *g*_m_ was assumed to be larger in C_4_ compared to C_3_ species, but most recent studies suggest that values for C_4_-*g*_m_ correspond to higher-end C_3_-*g*_m_ reports, and that C_4_-*g*_m_ reacts similarly to C_3_-*g*_m_ with regards to variation in factors such as leaf age and temperature (Barbour *et al.*, 2016; Osborn *et al.*, 2017; Ubierna *et al.*, 2017). If C_4_-*g*_m_ is lower than previously thought, that could affect derivations of other key parameters such as leakiness (ϕ, the proportion of C fixed by PEPC that subsequently leaks out the bundle-sheath cells). Leakiness cannot be directly measured and is commonly estimated from observations and models of ^13^C discrimination (Δ^13^C) (Farquhar, 1983; Farquhar and Cernusak, 2012). Historically, *g*_m_ is generally assumed to be infinite when solving for ϕ from Δ^13^C; however, this simplification and estimates of ϕ would be compromised if *g*_m_ is finite and low.

Mesophyll conductance has long been recognized as a significant limitation for C_3_ photosynthesis (Evans, 1983; Evans *et al.*, 1986; Evans and Terashima, 1988), limiting photosynthesis as much as stomatal conductance (Warren, 2008). It is unclear if *g*_m_ limits C_4_ photosynthesis as the reduction of photorespiration achieved by the CO_2_-concentrating mechanism saturates C_4_ photosynthesis at ambient *p*CO_2_. If *g*_m_ were to limit C_4_ photosynthesis, it would likely only be at very low *p*CO_2_. However, not much is known about the variation of C_4_-*g*_m_ with *p*CO_2_. In the C_4_ grass *Setaria viridis*, *g*_m_ derived with the ^18^O discrimination (Δ^18^O) method increased as *p*CO_2_ decreased, although the variation was not significant (Osborn *et al.*, 2017). Some reports have shown that in C_3_ species *g*_m_ increases with short-term exposure to decreasing *p*CO_2_ (Bongi and Loreto, 1989; Loreto *et al.*, 1992; Flexas *et al.*, 2007, 2008; Hassiotou *et al.*, 2009; Bunce, 2010; Douthe *et al.*, 2011; Tazoe *et al.*, 2011). However, others have suggested that C_3_-*g*_m_ is insensitive to changes in *p*CO_2_ (Loreto *et al.*, 1992; Tazoe *et al.*, 2009). It has been hypothesized that the observed C_3_-*g*_m_ response to *p*CO_2_ might result from a significant chloroplast resistance (Tholen and Zhu, 2011; Tholen *et al.*, 2012) or artifacts in the calculations (Gu and Sun, 2014).

In C_4_ plants, *g*_m_ has been estimated with the Δ^18^O method (Gillon and Yakir, 2000*a*, 2000*b*; Barbour *et al.*, 2016; Osborn *et al.*, 2017; Ubierna *et al.*, 2017) and the *in vitro* maximal PEP carboxylation rate (*V*_pmax_) method (Ubierna *et al.*, 2017). The latter method solves for the *p*CO_2_ in the mesophyll cells (*C*_m_) needed to simultaneously match modeled and measured rates of CO_2_ assimilation and Δ^13^C when the models are parameterized with *in vitro V*_pmax_, as determined in a crude leaf extract. Values derived for *g*_m_ with the Δ^18^O and *in vitro V*_pmax_ methods were similar in two C_4_ species measured over a range of temperatures (Ubierna *et al.*, 2017). The *in vitro V*_pmax_ method also allows the implementation of two modeling alternatives: carbonic anhydrase (CA)-saturated and CA-limited. They differ in the calculation of PEP carboxylation rate as a function of CO_2_ or HCO_3_^–^ for the CA-saturated and -limited scenarios, respectively. Ubierna *et al.* (2017) found no difference between CA-limited and CA-saturated estimates of *g*_m_ at ambient *p*CO_2_, but CA limitation is expected at low *p*CO_2_.

In this study, we calculated *g*_m_ using the *in vitro V*_pmax_ method across a range of *p*CO_2_ in two C_4_ grasses, one economically important (*Zea mays*) and the other the adopted model system for studying C_4_ photosynthesis (*S. viridis*). Measurements were performed at three temperatures (10, 25, and 40 °C) in *Setaria* and at 25 °C in *Zea*. Our objectives were to: (1) describe the response of C_4_-*g*_m_ to short-term variation in *p*CO_2_; (2) evaluate the impact of disequilibrium between CO_2_ and HCO_3_^–^ at a range of *p*CO_2_ and temperatures; (3) investigate if *g*_m_ represents a limitation to C_4_ photosynthesis across *p*CO_2_; and (4) assess the impact of finite *g*_m_ on ϕ calculations.

## Materials and methods

### Plant material

Seeds of *Z. mays* (var. Trucker’s Favorite, Victory Seed Company, Oregon, USA) were grown in a greenhouse supplemented with artificial lighting at the School of Biological Sciences at Washington State University, Pullman, WA (USA) during August to October 2011. Seeds of *S. viridis* (A-010) were grown in a controlled environment growth chamber (Enconair Ecological GC-16) in 2013. Plants used for measurements were 4 and 6 weeks old for *Zea* and *Setaria*, respectively. *Zea* was fertilized with 17-3-6 NPK and weekly additions of 4 g l^–1^ solution of 10% Fe-DPTA (Sprint 330, Becker Underwood, IA, USA). *Setaria* was treated weekly with Peters 20-20-20 (J. R. Peters, Inc., Allentown, PA, USA). For all plants, the photon flux density was ≥500 μmol m^–2^ s^–1^, the day length was 14 h, and the temperature was 25–28/20–25 °C for day/night.

### Coupled gas exchange and isoflux measurements

The system used for measurements has been described in detail in Ubierna *et al.* (2013, 2017). Briefly, a LI-6400XT open gas exchange system assembled with a 6400-22L conifer chamber fitted with a LI-6400–18 RGB light source (Li-Cor, Lincoln, NE, USA) was coupled with a tunable-diode laser absorption spectroscope (TDLAS, TGA 200A, Campbell Scientific, Inc. Logan, UT, USA). The entire gas exchange system was placed in a growing cabinet (Percival Scientific, Perry, IA), where the temperature was varied to match leaf temperature (*T*_L_) settings. The TDLAS data were calibrated with the concentration series method (Tazoe *et al.*, 2011; Ubierna *et al.*, 2013) using two calibration gases, one measured at different [CO_2_] that spanned the gas exchange reference and sample lines. Each measurement cycle included five to seven TDLAS sequences of zero air, calibration gases, reference, and sample lines measured for 40 s each. Data from the last three sequences were averaged and used for calculations.

Young fully-expanded leaves of *Setaria* and *Zea* were acclimated for ~1 h with chamber conditions of *C*_a_ (ambient CO_2_ supply to the chamber) ≈ 35 Pa, 21% O_2_, and photosynthetically active radiation (PAR) =2000 μmol m^–2^ s^–1^. Then, *C*_a_ was varied in steps, and gas and ^13^C isotopic exchange were measured simultaneously. In *Setaria* (*n*=4) *C*_a_ was set at 5, 7, 10, 12, 14, 19, 28, 38, 56, and 93 Pa, and measurements were performed at *T*_L_=10, 25 and 40 °C. In *Zea* (*n*=3), *C*_a_ was set at 9, 14, 19, 35, 56, 84, and 112 Pa, and *T*_L_=25 °C. In both species the measurements were performed in the sequence ambient – low – ambient – high *p*CO_2_.

### Enzyme-limited C_4_ photosynthesis model for CA-limited or CA-saturated conditions

The enzyme-limited C_4_ photosynthesis rate is (von Caemmerer, 2000):

$$A=\frac{-b-\sqrt{b^{2}-4ac}}{2a},$$ Eqn 1

where:

$$a=1-\frac{\alpha }{u_{\text{oc}}}\frac{K_{c}}{K_{o}},$$ Eqn 2

$$b=-\left[V_{p}-R_{m}+g_{bs}C_{m}+V_{cmax}-R_{d}+g_{bs}K_{c}\left(1+\frac{O_{\text{m}}}{K_{o}}\right)+\frac{\alpha }{u_{\text{oc}}}\left({\text{γ}}^{*}V_{cmax}+R_{d}\frac{K_{c}}{K_{o}}\right)\right],$$ Eqn 3

$$c=\left(V_{cmax}-R_{d}\right)\left(V_{p}-R_{m}+g_{bs}C_{m}\right)-V_{cmax}g_{bs}{\text{γ}}^{*}O_{m}-R_{d}g_{bs}K_{c}\left(1+\frac{O_{\text{m}}}{K_{o}}\right),$$ Eqn 4

where α (= 0) is the fraction of PSII activity in the bundle-sheath cells (von Caemmerer, 2000); *u*_oc_ is the ratio of O_2_ and CO_2_ diffusivities and solubilities, 0.047 at 25 °C but variable with temperature (Yin *et al.*, 2016); *g*_bs_ is the bundle-sheath conductance, 0.0164 μmol m^–2^ s^–1^ Pa^-1^ (Ubierna *et al.*, 2013) or variable; *O*_m_ is the O_2_ partial pressure in the mesophyll (19.5 kPa, which corresponds to 21%); *R*_d_ is the non-photorespiratory CO_2_ released in the dark, assumed to equal measured rates of dark respiration after 30 min of dark adaptation, which at 25 °C were 1.89 and 1.06 μmol m^–2^ s^–1^ in *Zea* and *Setaria*, respectively, but were also measured at each temperature; *R*_m_ is the mesophyll mitochondrial respiration rate, *R*_m_=0.5*R*_d_ (von Caemmerer, 2000); γ* is half of the reciprocal of Rubisco specificity, and equals 0.5/*S*_C/O_ (von Caemmerer, 2000), where *S*_C/O_ is the Rubisco CO_2_/O_2_ specificity. *K*_C_ and *K*_O_ are the Michaelis–Menten constants of Rubisco for CO_2_ and O_2_, respectively. *S*_C/O_, *K*_C_, and *K*_O_ were determined *in vitro* at 25 °C in *Zea* (*S*_C/O_=2147 Pa Pa^–1^, *K*_C_=96 Pa, *K*_O_=49 683 Pa; R.A. Boyd, Washington State University, pers. comm.) and *Setaria* (*S*_C/O_=1310 Pa Pa^–1^, *K*_C_=121 Pa, *K*_O_=29 200 Pa; Boyd *et al.*, 2015). Their values at different temperatures were obtained using the temperature functions of Boyd *et al.* (2015). For *V*_cmax_ (maximal Rubisco carboxylation rate) we used *in vivo* values calculated as described in Ubierna *et al.* (2017) or as specified otherwise. The calculation of *C*_m_ (*p*CO_2_ in the mesophyll cells) will be discussed subsequently.

CA-saturated and CA-limited models differ as follows.

(1) The calculation of PEP carboxylation rate (*V*_p_):

$$V_{p}=\left\{\begin{array}{c} CA\;saturated\to \frac{C_{m}V_{pmax}}{C_{m}+K_{p}}\;\\ CA\;limited\to \frac{\left[HCO_{3}^{-}\right]V_{pmax}}{\left[HCO_{3}^{-}\right]+K_{p}}\; \end{array}\right\},$$ Eqn 5

where the maximal PEP carboxylation rate (*V*_pmax_) was measured *in vitro* at 25 °C in *Zea* (184 μmol m^–2^ s^–1^, R. A. Boyd, pers. comm.) and in *Setaria* (450 μmol m^–2^ s^–1^, Boyd *et al.* 2015) and varied with temperature as described in Boyd *et al.* (2015). For all species, the Michaelis–Menten constant of PEPC for CO_2_ (*K*_P_) was modeled with the temperature response and value at 25 **°**C (60.5 μM HCO_3_^–^) from Boyd *et al.* (2015). The [HCO_3_^–^] was calculated as previously discussed (Jenkins *et al.*, 1989; Hatch and Burnell, 1990; Boyd *et al.*, 2015): for details see Ubierna *et al.* (2017).

If the rate of PEP regeneration is limiting, then *V*_p_ is (von Caemmerer, 2000):

$$V_{p}=\text{min}\left(V_{\text{p}}\;calculated\;with\;Eqn\;5,\;V_{pr}\right),$$ Eqn 6

where *V*_pr_ is the PEP regeneration rate (Peisker, 1986; Peisker and Henderson, 1992). We arbitrarily set *V*_pr_ to 64 and 59 μmol m^–2^ s^–1^ in *Setaria* and *Zea*, respectively, which corresponded to twice the maximum measured net assimilation rate, *A*.

(2) The calculation of the ratio *V*_p_/*V*_h_, where *V*_h_ is hydration rate:

$$\frac{V_{p}}{V_{h}}=\left\{\begin{array}{c} CA\;saturated\to 0\;\\ CA\;limited\to \frac{V_{p}}{C_{\text{m}}K_{CA}}\; \end{array}\right\},$$ Eqn 7

where *K*_CA_ is the rate constant of CA for CO_2_, that at 25 °C was 65.5 and 124 μmol m^–2^ s^–1^ Pa^–1^ in *Zea* and *Setaria*, respectively (R.A. Boyd pers. comm., Boyd *et al.*, 2015), varying with temperature as described in Boyd *et al.* (2015).

### Measurements and models of discrimination

The observed photosynthetic discrimination against ^13^C ($\Delta_{obs}^{13}$) is calculated as (Evans *et al.*, 1986):

$$\Delta_{obs}^{13}=\frac{\frac{C_{in}}{C_{in}-C_{out}}\left(\delta_{out}-\delta_{in}\right)}{1+\delta_{out}-\frac{C_{in}}{C_{in}-C_{out}}\left(\delta_{out}-\delta_{in}\right)},$$ Eqn 8

where *C* and δ are the ^12^CO_2_ mol fraction and the δ^13^C of the CO_2_, respectively, in dry air in and out the chamber.

The theoretical model for Δ^13^C is (Farquhar and Cernusak, 2012):

$$\Delta_{theo}^{13}=\frac{1}{1-t}\left[a_{b}\frac{C_{a}-C_{L}}{C_{a}}+a_{s}\frac{C_{L}-C_{i}}{C_{a}}\right]+\frac{1+t}{1-t}\left[a_{m}\frac{C_{i}-C_{m}}{C_{a}}+\frac{b_{4}+\phi \left(\frac{b_{3}C_{bs}}{C_{bs}-C_{m}}\;-\;s\right)}{1+\frac{\phi C_{m}}{C_{bs}-C_{m}}}\frac{C_{m}}{C_{a}}\right].$$ Eqn 9

Values and calculations of the variables included in this equation have been discussed before (i.e. Ubierna *et al.*, 2017) and can also be found in Supplementary Methods S1 at *JXB* online.

### Calculation of mesophyll conductance (*g*_m_)

Following Fick’s law of diffusion:

$$g_{\text{m}}=\frac{A}{C_{\text{i}}-C_{\text{m}}},$$ Eqn 10

where the *C*_m_ is calculated for two case scenarios, CA-saturated and CA–limited, resulting in CA-sat *g*_m_ and CA-lim *g*_m_ values. In both cases, *C*_m_ is derived with the *in vitro V*_pmax_ method as the *C*_m_ that needs to be combined with *in vitro V*_pmax_ to match measurements and predictions of *A* and Δ^13^C (Eqns 1, 9); details on these calculations have been provided in Ubierna *et al.* (2017). The CA-sat and CA-lim options are introduced through the calculation of *V*_p_ and *V*_p_/*V*_h_ (Eqns 5–7).

### Limitations to photosynthesis

To calculate the limitation on CO_2_ assimilation by either finite stomatal conductance (*L*_s_), by mesophyll conductance (*L*_m_), or by carbonic anhydrase (*L*_CA_), we adapted to C_4_ photosynthesis an approach previously used for C_3_ photosynthesis. This compares *A* when all conductances are finite with the *A* estimated assuming that the conductance related with the limitation of interest is infinite (Farquhar and Sharkey, 1982; Warren *et al.*, 2003). In all cases *A* was calculated with Eqn 1 and assuming:

(a) *A*_all_ (expected *A* with all limitations, ≈ measured photosynthetic rate): finite *g*_s_ and *g*_m_, CA-lim model.
(b) *A*_s_ (expected *A* if there were no stomatal limitations): infinite *g*_s_ (*C*_i_=*C*_a_), finite *g*_m_, CA-lim model.
(c) *A*_m_ (expected *A* if there were no mesophyll limitations): infinite *g*_m_ (*C*_m_=*C*_i_), *g*_s_ as measured, CA-lim model.
(d) *A*_CA_ (expected *A* if there were no CA limitations): *g*_s_ as measured, *g*_m_ finite, CA-sat model.

Then *L*_s_, *L*_m_, and *L*_CA_ were calculated as:

$$L_{\text{s}}=100\times \frac{A_{\text{s}}-A_{\text{all}}}{A_{\text{s}}},$$ Eqn 11

$$L_{\text{m}}=100\times \frac{A_{\text{m}}-A_{\text{all}}}{A_{\text{m}}},$$ Eqn 12

$$L_{\text{CA}}=100\times \frac{A_{\text{CA}}-A_{\text{all}}}{A_{\text{CA}}}.$$ Eqn 13

### Calculation of leakiness (ϕ)

The C_4_ photosynthesis model (von Caemmerer, 2000) calculates ϕ as:

$$\phi =\frac{g_{\text{bs}}\left(C_{\text{bs}}-C_{\text{m}}\right)}{V_{\text{p}}},$$ Eqn 14

where *C*_bs_, the *p*CO_2_ in the bundle-sheath cells, is (von Caemmerer, 2000):

$$C_{\text{bs}}=\frac{{\text{γ}}^{*}O_{\text{s}}+K_{\text{C}}\left(1+\frac{O_{\text{s}}}{K_{\text{o}}}\right)\left(\frac{A+R_{\text{d}}}{V_{\text{cmax}}}\right)}{1-\frac{A+R_{\text{d}}}{V_{\text{cmax}}}}=C_{\text{m}}+\frac{V_{\text{p}}-A-R_{\text{m}}}{g_{\text{bs}}},$$ Eqn 15

where *O*_s_ is the O_2_ partial pressure in the bundle-sheath cells.

From Δ^13^C (Eqn 9), ϕ is solved as:

$$\phi_{1}=\frac{C_{\text{bs}}-C_{\text{m}}}{C_{\text{m}}}\times \frac{\Delta_{obs}^{13}\left(1-t\right)C_{\text{a}}\;-\;\bar{a}\left(C_{\text{a}}-C_{i}\right)\;-\;a_{\text{m}}\left(C_{\text{i}}-C_{\text{m}}\right)\left(1+t\right)\;-\;b_{4}\left(1+t\right)C_{\text{m}}}{\left(1+t\right)\left[b_{3}C_{\text{bs}}\;-\;s\left(C_{\text{bs}}-C_{\text{m}}\right)\;+\;a_{\text{m}}\left(C_{\text{i}}-C_{\text{m}}\right)\right]\;+\;\bar{a}\left(C_{\text{a}}-C_{\text{i}}\right)\;-\;C_{\text{a}}\Delta_{obs}^{13}\left(1-t\right)}$$ Eqn 16

where *b*_3_ (combined effects of Rubisco fractionation, and fractionations associated with respiration and photorespiration) and *b*_4_ (combined fractionation during PEP carboxylation, hydration, and respiration) are calculated as (Farquhar, 1983; Cousins *et al.*, 2006):

$$b_{3}≅b_{3}^{´}-\frac{eR_{\text{d}}}{V_{\text{c}}}-\frac{0.5fV_{\text{o}}}{V_{\text{c}}},$$ Eqn 17

$$b_{4}=b_{4}^{´}\left(1-\frac{V_{\text{p}}}{V_{\text{h}}}\right)+(e_{\text{s}}+h)\frac{V_{\text{p}}}{V_{\text{h}}}-\frac{eR_{\text{m}}}{V_{\text{p}}}.$$ Eqn 18

A description of other variables included in Eqns 16–19 can be found in Supplementary Methods S1.

To evaluate the effect of *g*_m_ on calculations of ϕ we implemented four model scenarios, which differed in values for *g*_m_, calculation of *V*_p_, or constrains imposed. Model 1 used *in vitro V*_pmax_ and *g*_m_ finite and equal to the values for CA-lim *g*_m_ presented in the Results; Model 2 used *in vivo V*_pmax_ and *g*_m_ infinite; Model 3 was the same as Model 1 but the solution was only constrained by *A* and not Δ^13^C; and Model 4 was the same as Model 1 but with *V*_p_ calculated with Eqn 6, which introduces a PEP regeneration limitation. The *in vitro V*_pmax_ method calculates *g*_m_ by solving the system of two equations formed by the models of *A* and Δ^13^C. Therefore, once a solution is found, ϕ values calculated with either Eqn 14 or 16 are identical. This is the case for Models 1, 2, and 4; however, in Model 3, which is constrained only by *A*, ϕ was obtained only with Eqn 14. All four modeling scenarios described above used the CA-limited calculations (Eqns 5–7).

At ambient *p*CO_2_, ϕ was also calculated with a simplified equation derived from Δ^13^C assuming that *C*_bs_ is much larger than *C*_m_ and that hydration and assimilation fluxes are large (*V*_p_/*V*_h_≈0, and *V*_o_≈0, where *V*_o_ is oxygenation rate):

$$\phi_{2}=\;\frac{\frac{\Delta_{\text{obs}}\left(1-t\right)C_{\text{a}}\;-\;\bar{a}\left(C_{\text{a}}-C_{\text{i}}\right)}{1+t}\;-\;\;a_{\text{m}}C_{\text{i}}\;+\;C_{\text{m}}\left(a_{\text{m}}-\bar{b_{4}}\right)}{C_{\text{m}}\left(\bar{b_{3}}-s\right)},$$ Eqn 19

where $\bar{b_{3}}$ and $\bar{b_{4}}$ are (von Caemmerer *et al.*, 2014):

$$\bar{b_{3}}=b_{3}^{´\;}-\frac{eR_{\text{d}}}{A+R_{\text{d}}}+\frac{e0.5R_{\text{d}}}{A+0.5R_{\text{d}}},$$ Eqn 20

$$\bar{b_{4}}=b_{4}^{´\;}-\frac{e0.5R_{\text{d}}}{A+0.5R_{\text{d}}}.$$ Eqn 21

### Statistical analyses

Statistical analyses were performed using SAS v9.4 (SAS Institute Inc., Cary, NC, USA). Differences between CA-lim *g*_m_ and CA-sat *g*_m_ were investigated using *t*-tests (*H*_o_: CA-lim *g*_m_/CA-sat *g*_m_=1). The effect of CO_2_ supply on CA-lim *g*_m_ was analysed using repeated measurements ANOVA. Data were log-transformed to meet normality criteria. In *Setaria* we used PROC MIXED with: *plant* as the repeated measurement; *pCO*_*2*_, *temperature*, and their interaction as fixed effects; a covariance structure of compound symmetry; and we applied Kenward–Roger’s approximation to correct the denominator degrees of freedom (Arnau *et al.*, 2009). In *Zea*, we used PROC ANOVA with the statement REPEAT.

## Results

### *A*-*C*_i_ curves and observed ^13^C photosynthetic discrimination

**Fig. 1.**
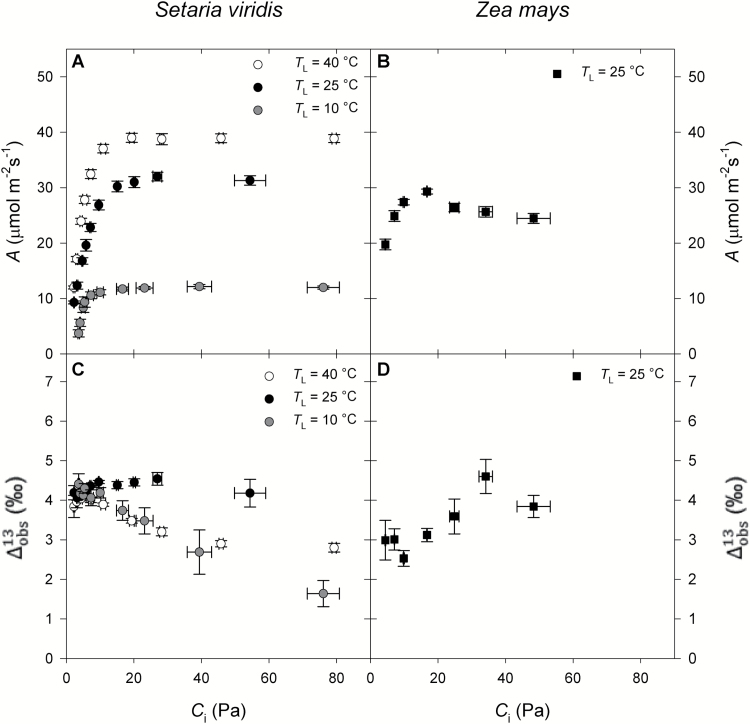
Responses of (A, B) photosynthetic rate (*A*) and (C, D) observed ^13^C photosynthetic discrimination ($\Delta_{obs}^{13}$) to variation in the CO_2_ partial pressure inside the leaf (*C*_i_) in *Setaria viridis* (circles) and *Zea mays* (squares). In *Setaria*, three leaf temperatures (*T*_L_) were measured: 40, 25, and 10 °C, as indicated in the key. Measurements in *Zea* were at *T*_L_=25 °C. Values are means ±SE; *n*=4 in *Setaria* and *n*=3 in *Zea*.

Under all leaf measurement temperatures (*T*_L_), the rate of net photosynthesis (*A*) in *Setaria* increased with *C*_i_ as the *p*CO_2_ supplied increased from ~5 Pa to ambient air values (~35 Pa) and then leveled off (Fig. 1A). At all *p*CO_2_, increasing *T*_L_ resulted in larger *A* (Fig. 1A). In *Zea*, *A* also increased with increasing *C*_i_ and reached a maximum at ambient air *p*CO_2_ before decreasing at higher *p*CO_2_ (Fig. 1B).

At ambient air *p*CO_2_ and 25 °C, $\Delta_{obs}^{13}$ was larger in *Setaria* (4.5 ± 0.1‰) than in *Zea* (3.1 ± 0.2‰) (Fig. 1C, D). In *Zea*, the $\Delta_{obs}^{13}$ was low at ambient air *p*CO_2_ and increased at lower or higher *C*_i_ (Fig. 1D). However, in *Setaria*, $\Delta_{obs}^{13}$ remained constant with *C*_i_ when *T*_L_=25 °C, but decreased as *C*_i_ increased both at 40 and 10 °C (Fig. 1C).

### Mesophyll conductance calculated assuming CA-saturated or CA-limited conditions

**Fig. 2.**
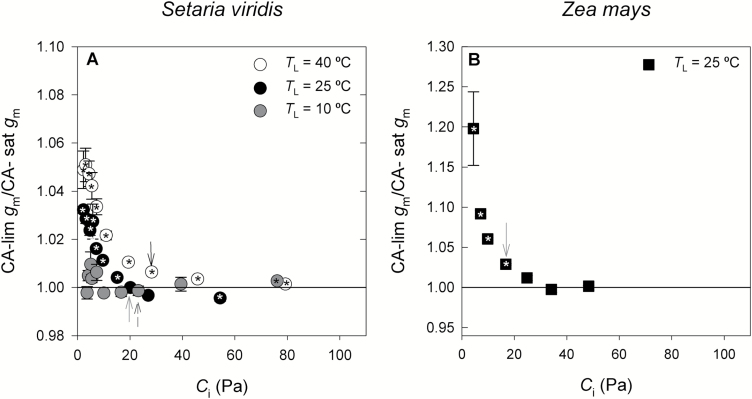
The ratio of carbonic anhydrase-limited mesophyll conductance (CA-lim *g*_m_) to CA-saturated *g*_m_ (CA-sat *g*_m_) at different *p*CO_2_ inside the leaf (*C*_i_) in (A) *Setaria viridis* (circles) and (B) *Zea mays* (squares). *Setaria* was measured at three leaf temperatures (*T*_L_): 40, 25, and 10 °C, as indicated in the key. *Zea* was measured at *T*_L_=25 °C. Values are means ±SE; *n*=4 in *Setaria* and *n*=3 in *Zea*. An asterisk inside a symbol indicates CA-lim *g*_m_/CA-sat *g*_m_ ≠ 1 with *P*<0.05. The arrows indicate the values at ambient *p*CO_2_ and at 40 °C (black arrow), 25 °C (grey arrow), and 10 °C (dashed arrow).

For both species and at all temperatures, the ratio CA-lim *g*_m_/CA-sat *g*_m_ ≈ 1 when *p*CO_2_ was above ambient (Fig. 2). As *p*CO_2_ decreased, CA-lim *g*_m_ became larger than CA-sat *g*_m_; the differences increased with temperature and were larger in *Zea* than in *Setaria*. In *Setaria*, CA-lim *g*_m_ and CA-sat *g*_m_ were significantly different (*P*<0.05) at all *p*CO_2_ at 40 °C, at all *p*CO_2_ except at ambient and the measurement just above ambient at 25 °C, and at the largest *p*CO_2_ at 10 °C (Fig. 2A). In *Zea*, CA-lim *g*_m_ and CA-sat *g*_m_ were significantly different (*P*<0.05) at all *p*CO_2_≤ambient air (Fig. 2B).

In *Setaria*, the under-estimation of *g*_m_ by ignoring the CA limitation was very small (maximum of 5%, CA-lim *g*_m_/CA-sat *g*_m_<1.1; Fig. 2A). However, in *Zea*, the CA-lim *g*_m_ calculated at the lowest *p*CO_2_ was 20 ± 8% larger than CA-sat *g*_m_ at 25 °C. Because CA limitation was relevant at low *p*CO_2_, for subsequent analyses we use the CA-lim *g*_m_ values for all species, temperatures, and *p*CO_2_.

### CO_2_ response of mesophyll conductance

**Fig. 3.**
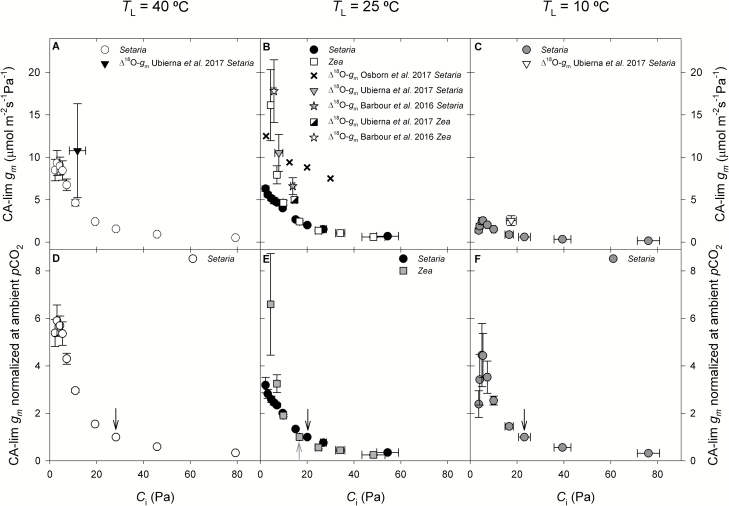
The response of carbonic anhydrase-limited mesophyll conductance (CA-lim *g*_m_) to changes in *p*CO_2_ inside the leaf (*C*_i_) in (A, C) *Setaria viridis* (circles) and (B) *Zea mays* (white squares). *Setaria* was measured at three leaf temperatures, as indicated at the top of the figure. *Zea* was measured at *T*_L_=25 °C. For comparison, the available literature reports for Δ^18^O-*g*_m_ for different species and temperatures are included, as indicated in the keys: Ubierna *et al.* (2017)*Setaria* measured at *T*_L_=40 °C, *T*_L_=25 °C, and *T*_L_=10 °C; Osborn *et al.* (2017)*Setaria* measured at *T*_L_=25 °C; Barbour *et al.* (2016)*Setaria* measured with block temperature of 30 °C; Ubierna *et al.* (2017)*Zea* measured at *T*_L_=25 °C; Barbour *et al.* (2016)*Zea* measured with block temperature of 30 °C. For all species and temperatures CA-lim *g*_m_ significantly varied with *p*CO_2_. (D–F) The CO_2_ response of normalized *g*_m_, calculated by dividing individual values by the *g*_m_ at ambient *p*CO_2_ at each temperature. Values are means ±SE; *n*=4 in *Setaria*, *n*=3 in *Zea*. The arrows indicate the values at ambient *p*CO_2_: black, *Setaria*; grey, *Zea*.

The CA-lim *g*_m_ significantly increased as *p*CO_2_ decreased in *Setaria* at all temperatures (*P*<0.0001) and in *Zea* at 25 °C (*P*<0.0004) (Fig. 3). At ambient *p*CO_2_ and 25 °C, CA-lim *g*_m_ values (mean±SE) were 2.00 ± 0.10 μmol m^–2^ s^–1^ Pa^–1^ in *Setaria*, and 2.43 ± 0.13 μmol m^–2^ s^–1^ Pa^–1^ in *Zea*. At the lowest *p*CO_2_ measured (~5–9 Pa) and 25 °C, the CA-lim *g*_m_ increased to 6.30 ± 0.32 and 16.20 ± 5.74 μmol m^–2^ s^–1^ Pa^–1^ in *Setaria* and *Zea*, respectively. Values for *C*_m_ across *C*_i_ can be found in Supplementary Fig. S1.

To compare the magnitude of the change in CA-lim *g*_m_ across species and temperatures, CA-lim *g*_m_ was normalized by dividing each value at a given temperature and *p*CO_2_ by CA-lim *g*_m_ at ambient *p*CO_2_ at that temperature (Fig. 3D–F). At 25 °C, the increase in CA-lim *g*_m_ with decreasing *p*CO_2_ was steeper in *Zea* than in *Setaria* (Fig. 3E). In *Setaria*, the *g*_m_*p*CO_2_ response was greatest at 40 °C and there was little difference between the 25 and 10 °C curves.

### Limitations to photosynthesis

**Fig. 4.**
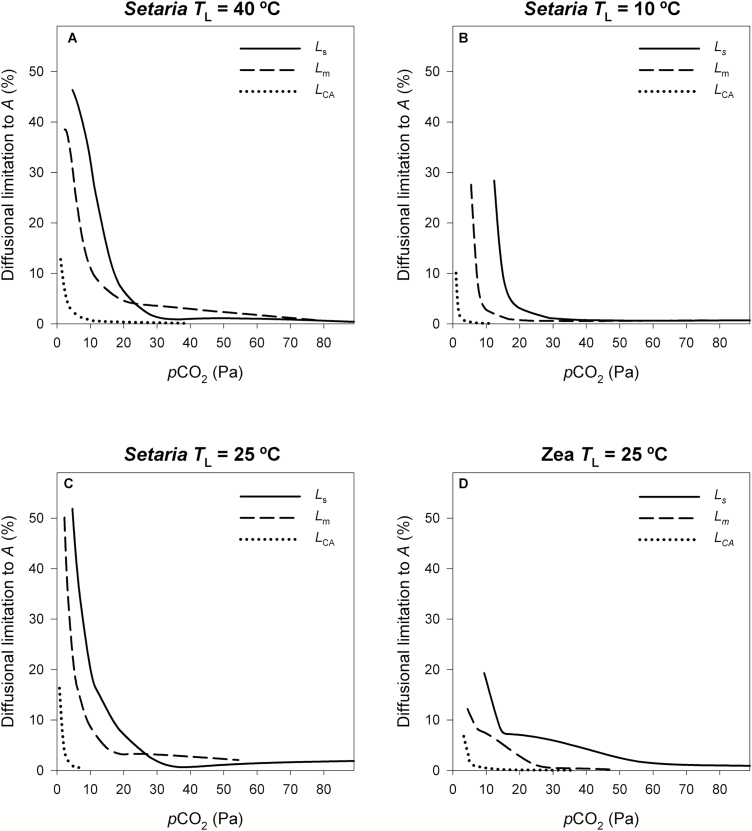
Diffusional limitation to photosynthetic rate (*A*) imposed by stomatal resistance (*L*_s_, Eqn 11, solid line), mesophyll resistance (*L*_m_, Eqn 12, dashed line), and carbonic anhydrase (*L*_CA_, Eqn 13, dotted line) as a function of the CO_2_ supply (*p*CO_2_) available for each (*C*_a_, *C*_i_, and *C*_m_ for *L*_s_, *L*_m_, and *L*_CA_, respectively). (A) *Setaria viridis* at *T*_L_=40 °C, (B) *Setaria viridis* at *T*_L_=10 °C, (C) *Setaria viridis* at *T*_L_=25 °C, and (D) *Zea mays* at *T*_L_=25 °C.

At elevated *p*CO_2_ assimilation rate was not limited by diffusion or substrate availability, as indicated by *L*_s_, *L*_m_, and *L*_CA_ ≈ 0% for both species and all temperatures (Fig. 4). However, below ambient *p*CO_2_, the diffusional limitation to *A* increased exponentially with decreasing *p*CO_2_. The data in Fig. 4 show the different limitations as a function of the amount of substrate available: *C*_a_, *C*_i_, and *C*_m_ for *L*_s_, *L*_m_, and *L*_CA_, respectively. In *Setaria*, diffusional limitations were lower at 10 °C than at any other temperature. Comparing *Zea* and *Setaria* at 25 °C, they had similar *L*_s_ but *L*_m_ was larger in *Setaria* than in *Zea*. For example, when *C*_a_=9 Pa, *L*_s_=23% and 19% in *Setaria* and *Zea*, respectively. The corresponding *C*_i_ at this *C*_a_ was 5 Pa for both species, whereas *L*_m_ was almost double in *Setaria* (23%) compared to *Zea* (12%) (Fig. 4C, D). In both species, *L*_CA_ was small in comparison with *L*_s_ and *L*_m_, and rapidly decreased below 5% as *p*CO_2_ increased.

### Leakiness (ϕ)

**Fig. 5.**
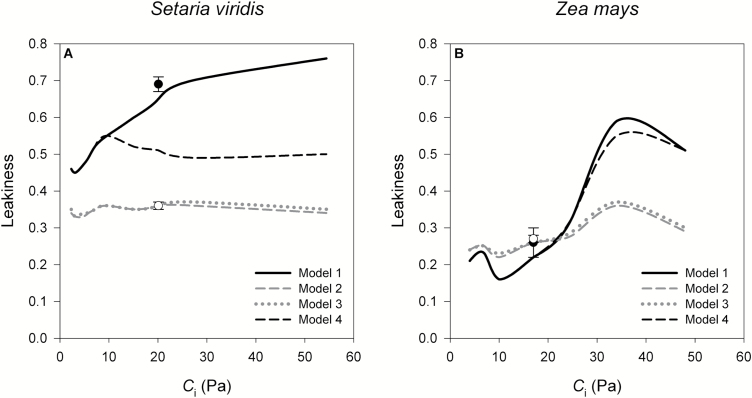
Effect of different parameterizations of models of photosynthesis in the calculation of leakiness (ϕ) in (A) *Setaria virids* and (B) *Zea mays* at 25 °C and over a range of *p*CO_2_ inside the leaf intercellular spaces (*C*_i_). Model 1 (solid black line) uses *in vitro V*_pmax_ and *g*_m_ finite and equal to the values presented in Fig. 3; Model 2 (dashed grey line) uses *in vivo V*_pmax_ (which is variable with *p*CO_2_, see Supplementary Fig. S2) and *g*_m_ infinite; Model 3 (dotted grey line) uses the same as Model 1 but the solution was only constrained by *A* and not Δ^13^C; Model 4 (dashed black line) uses the same as Model 1 but introducing *V*_pr_ (= 64 and 59 μmol m^–2^ s^–1^ in *Setaria* and *Zea*, respectively) in the calculation of *V*_p_ (Eqn 6). The rest of the variables included in these models were calculated as explained in the Methods section: values for some of them can be found in Supplementary Fig. S2. In Models 1, 2, and 4, ϕ was calculated with Eqns 14 or 16 (same result) and in Model 3, ϕ was calculated with Eqn 14. The symbols indicate the value of ϕ at ambient air *p*CO_2_ calculated with the simplified Eqn 19 assuming either *g*_m_ finite (solid symbols) or infinite (clear symbols). Values are means ±SE; *n*=4 in *Setaria*, *n*=3 in *Zea*.

Values of ϕ across *p*CO_2_ for *Setaria* and *Zea* at 25 °C calculated under different modeling assumptions are shown in Fig. 5. When *g*_m_ was finite and variable with *p*CO_2_ (Model 1), ϕ increased from low to high *p*CO_2_, with a range of 0.16–0.59 in *Zea* and 0.45–0.76 in *Setaria*. Assuming that *g*_m_ was infinite and *V*_pmax_ variable with *p*CO_2_ (Model 2) removed the *p*CO_2_ response of ϕ and generally decreased ϕ at all *p*CO_2_ in *Setaria*, but only at large *p*CO_2_ in *Zea*. Model 3 resulted in nearly identical ϕ to Model 2 using the same finite *g*_m_ as Model 1 but with the solution constrained by only the photosynthesis model. However, this scenario failed to predict $\Delta_{obs}^{13}$ (see Supplementary Fig. S2). Imposing a PEP regeneration rate (*V*_pr_) limitation of 64 and 59 μmol m^–2^ s^–1^ in *Setaria* and *Zea*, respectively (Model 4), decreased ϕ compared to the results with Model 1 in *Setaria* but resulted in no change in *Zea*. Interestingly, at *p*CO_2_ ≤ambient air, values for ϕ were similar across models in *Zea*, but they differed in *Setaria*. The values of *V*_pmax_, *V*_cmax_, *V*_p_, *V*_c_, *C*_bs_, and *g*_bs_ used in these four models are reported in Supplementary Fig. S2.

For comparison we also present ϕ at ambient *p*CO_2_ calculated with the simplified Eqn 19 and assuming either *g*_m_ finite or infinite. For both species, ϕ calculated with Eqn 19 was not different to values obtained with the complete Eqn 16 when *g*_m_ was finite (compare black lines and black symbols in Fig. 5) and when *g*_m_ was infinite (compare grey dashed line and clear symbols).

## Discussion

### Calculation of mesophyll conductance and model parameterization

Mesophyll conductance (*g*_m_) was derived with the *in vitro V*_pmax_ method (Ubierna *et al.*, 2017). Estimations of *g*_m_ with this method were similar to Δ^18^O-*g*_m_ across temperatures (Ubierna *et al.*, 2017) and across *p*CO_2_ (Kolbe and Cousins, 2018). Potential errors in *g*_m_ originating from inaccurate model parameterization of the *in vitro V*_pmax_ method were tested with a sensitivity analysis using *Setaria* data at three temperatures and across *p*CO_2_ (see Supplementary Fig. S3). Halving *in vitro V*_pmax_ increased *g*_m_ by <20% at large *p*CO_2_ and almost doubled it at low *p*CO_2_ and high temperature. Alternatively, doubling *in vitro V*_pmax_ decreased *g*_m_ by <15% at all *p*CO_2_ and temperatures ( Supplementary Fig. S3J–L). This demonstrates that uncertainties in *in vitro V*_pmax_ affect absolute values of *g*_m_, but not the trend of increasing *g*_m_ with decreasing *p*CO_2_. The sensitivity analysis also demonstrated that variations up to ±50% in *K*_P_, *K*_C_, or *K*_CA_ resulted in negligible (when *p*CO_2_ ≥ambient) or small (at low *p*CO_2_) errors in *g*_m_ calculations at any temperature (Supplementary Fig. S3A–I) and did not affect the observed trend of *g*_m_ with *p*CO_2_.

In C_3_ plants, it has been suggested that large *g*_m_ values reported for low *p*CO_2_ might be an artifact of uncertainties in parameters such as *R*_d_, Γ*, and *b*′_3_ (Gu and Sun, 2014). The simulations with different values for *R*_d_ (see Supplementary Fig. S4A, B) or *b*′_3_ (Supplementary Fig. S4C, D) resulted in variations in *g*_m_ of <6% and did not affect the trend of increasing *g*_m_ with decreasing *p*CO_2_. Ubierna *et al.* (2017) demonstrated that *g*_m_ is largely independent of values of *g*_bs_ or *V*_cmax_ and this is also illustrated in Supplementary Fig. S2.

### CA-limited versus CA-saturated models to estimate *g*_m_

The substrate for the initial carboxylation by PEPC is HCO_3_^–^ and not CO_2_. However, *V*_p_ is often calculated in terms of CO_2_, because the hydration of CO_2_ (*V*_h_) generally happens very fast when catalysed by CA (Stryer, 1988). We refer to this case as the CA-saturated model. In contrast, the CA-limited model calculates *V*_p_ as a function of HCO_3_^–^. The value of HCO_3_^–^ is calculated with *C*_m_, *V*_h_, *V*_pmax_, and a series of rate constants (see Ubierna *et al.*, 2017, for details). Producing the same *V*_p_ with the CA-limited and the CA-saturated calculations requires larger *C*_m_ for the former than the latter, and the difference could potentially be large if *V*_h_ is low. Subsequently, neglecting the hydration step, as in the CA-saturated calculations, can result in under-estimation of *C*_m_ and *g*_m_. The terminology CA-saturated or -limited refers to the modeling of *V*_p_ and how this affects the calculated *C*_m_ value, but it does not imply different roles of CA in the photosynthetic process. Ubierna *et al.* (2017) found no difference between CA-sat *g*_m_ and CA-lim *g*_m_ at ambient *p*CO_2_; however, the aim here is to compare these calculations for a range of *p*CO_2_.

In both species and at all temperatures, the difference between CA-sat *g*_m_ and CA-lim *g*_m_ was negligible for *p*CO_2_ >ambient (Fig. 2). However, as *p*CO_2_ decreased, CA-lim *g*_m_ became larger than CA-sat *g*_m_, especially at high temperatures and in *Zea*. In this species ignoring the hydration step resulted in under-estimating *g*_m_ by as much as 20%, whereas in *Setaria* the under-estimation was <5%.

The larger differences at high temperatures can be explained by the temperature response of *K*_CA_, which increases from 10 to 30 °C but plateaus above that (Boyd *et al.*, 2015). Species differences can be explained by different *K*_CA_ values and CO_2_ availability to CA. Firstly, *K*_CA_ in *Setaria* (124 μmol m^–2^ s^–1^ Pa^–1^) was double the value for *Zea* (65.5 μmol m^–2^ s^–1^ Pa^–1^). Below ambient *p*CO_2_, *Setaria* and *Zea* had similar *A*, *g*_s_, and *C*_i_. Sustaining similar *A* in these two species requires larger *C*_m_ in *Zea* than in *Setaria* because of the lower *in vitro V*_pmax_ value in the former (184 μmol m^–2^ s^–1^) versus the latter (450 μmol m^–2^ s^–1^). Therefore, in *Zea* the lower *K*_CA_ and *in vitro V*_pmax_ was counterbalanced by increased CO_2_ availability to CA through higher *g*_m_. Osborn *et al.* (2017) also suggested large *g*_m_ as a mechanism to increase CO_2_ assimilation rate at low *p*CO_2_.

At low *p*CO_2_ or in species with low *K*_CA_, ignoring the hydration step results in under-estimation of *g*_m_. However, the error is insignificant at *p*CO_2_ above ambient or in species with large *K*_CA_, such as *Setaria*. The hydration step should be included for accurate determination of *g*_m_ at low *p*CO_2_ in species with low *K*_CA_ and/or high *A*, such as C_4_ grasses (Cousins *et al.*, 2008), especially at high temperatures.

### Values for CA-lim *g*_m_ and variation with *p*CO_2_

Across *p*CO_2_ and temperatures, CA-lim *g*_m_ ranged from 0.6 ± 0.1 to 9.3 ± 1.5 μmol m^–2^ s^–1^ Pa^–1^ in *Setaria*, and 0.6 ± 0.1 to 16.2 ± 5.7 μmol m^–2^ s^–1^ Pa^–1^ in *Zea* (Fig. 3). In *Zea*, photosynthetic rate declined above ambient *p*CO_2_, indicating deactivation at low *C*_i_ that did not fully recover when *p*CO_2_ supply was returned to ambient levels (Fig. 1B). This could have introduced some bias in the CA-lim *g*_m_ values calculated at high *p*CO_2_. Nevertheless, the CA-lim *g*_m_ values were used at *p*CO_2_ ≤ambient, because above ambient, photosynthesis was not restricted by diffusional limitations (Fig. 4).

To validate CA-lim *g*_m_ values, they were compared with literature reports for the same species obtained with the alternative Δ^18^O method (Barbour *et al.*, 2016; Osborn *et al.*, 2017; Ubierna *et al.*, 2017; Fig. 3). In *Zea*, there was a good agreement between Δ^18^O-*g*_m_ (Barbour *et al.*, 2016; Ubierna *et al.*, 2017) and CA-lim *g*_m_ (Fig. 3B). A recent study in *Zea* by Kolbe and Cousins (2018) also found agreement between Δ^18^O-*g*_m_ and *in vitro V*_pmax_*g*_m_ across a range of *p*CO_2_, although both estimations of *g*_m_ deviated at very low *p*CO_2_. In *Setaria*, Δ^18^O-*g*_m_ (Barbour *et al.*, 2016; Osborn *et al.*, 2017; Ubierna *et al.*, 2017) was larger than our CA-lim *g*_m_ results (Fig. 3A–C). This discrepancy could have originated if *in vitro V*_pmax_ was over-estimated, and more studies exploring *g*_m_ variation and assessing the impacts of the method are needed.

In *Zea* at 25 °C and in *Setaria* at three temperatures, the CA lim-*g*_m_ increased with short-term exposure to decreasing *p*CO_2_. Increasing *g*_m_ with decreasing *p*CO_2_ has also been observed in C_3_ species (Bongi and Loreto, 1989; Loreto *et al.*, 1992; Flexas *et al.*, 2007, 2008; Hassiotou *et al.*, 2009; Bunce, 2010; Douthe *et al.*, 2011; Tazoe *et al.*, 2011), although there are also a few studies that have concluded there is no change (Loreto *et al.*, 1992; Tazoe *et al.*, 2009). There are only two studies that have presented C_4_-*g*_m_ across *p*CO_2_. In Osborn *et al.* (2017), Δ^18^O-*g*_m_ values for *Setaria* increased with decreasing *p*CO_2_ but the trend was not significant. In *Zea*, Kolbe and Cousins (2018) found a significant increase in Δ^18^O-*g*_m_ with decreasing *p*CO_2_.

The initial slope of an *A*-*C*_i_ curve can be modified with either *C*_m_ (*g*_m_) or *V*_pmax_ (see Supplementary Fig. S5). Therefore, there may be a value for *V*_pmax_ that would cancel out the trend in CA-lim *g*_m_. However, this is not the case if *V*_pmax_ is independent of *p*CO_2_, and cancelling the observed trend in CA-lim *g*_m_ would require *V*_pmax_ to decrease with increasing *p*CO_2_ (Supplementary Fig. S6). There is evidence showing that CO_2_ levels affect the phosphorylation state of PEPC and PEPCK, and therefore variation of *in vivo V*_pmax_ across *p*CO_2_ could be expected (Bailey *et al.*, 2007). However, the CO_2_ response of photosynthetic rate was found to be no different between wild-type and transgenic plants with low PEPC phosphorylation (Furumoto *et al.*, 2007). Much of the post-translational modifications that presumably lower *V*_pmax_ would probably occur when CO_2_ is saturating and some other factor limits C_4_ photosynthesis. At ambient *p*CO_2_ and below it is generally thought, although not known, that PEPC is operating at *V*_pmax_. The fact that Δ^18^O-*g*_m_ data have demonstrated a similar trend of increasing *g*_m_ with decreasing *p*CO_2_ (Kolbe and Cousins, 2018) points to a constant *V*_pmax_ value. Nevertheless, if fast *in vivo* regulation of *V*_pmax_ occurs it could alter values and trends in *g*_m_. In reality, there might be a combination of both fluctuations in *g*_m_ and *V*_pmax_ in response to short-term variation in *p*CO_2_. Future work should investigate *in vivo* regulation of *V*_pmax_ and its impact on *g*_m_ calculations.

### Limitation to photosynthesis at low *p*CO_2_

C_4_ photosynthesis saturates at ambient *p*CO_2_ and *A* was not limited by diffusion, as indicated by *L*_s_, *L*_m_, and *L*_CA_ ≈ 0% for both species and all temperatures (Fig. 4). However, below ambient air *p*CO_2_, diffusional limitations constrained CO_2_ assimilation and increased exponentially with decreasing *p*CO_2_. As shown in Fig. 1 and Supplementary Fig. S5, in both species the CO_2_ responsive part of the *A*-*C*_i_ curve corresponded to *C*_i_ below ~10 Pa. This raises the question of whether C_4_ plants operate below this threshold. In laboratory experiments, high irradiance and N fertilization shifted the operational *C*_i_ down to the CO_2_ responsive part of the *A*-*C*_i_ curve (Ghannoum *et al.*, 1997; Ghannoum and Conroy, 1998). Additionally, moderate water stress decreased *C*_i_ in several C_4_ species, although under severe drought declines in *A* precluded *C*_i_ from getting very low (Ghannoum, 2009, and references herein). Under ambient air *p*CO_2_, *C*_i_<11 Pa were reported for *Zea* grown in FACE-type experiments (Leakey *et al.*, 2004; Markelz *et al.*, 2011), and *Sorghum bicolor* grown in an open field reached *C*_i_/*C*_a_=0.2 after two consecutive water-stress cycles (Steduto *et al.*, 1997). Therefore, under certain growth conditions, CO_2_ availability may limit C_4_ photosynthesis.

Interestingly, *Setaria* and *Zea* displayed different behavior at low *p*CO_2_. At low *p*CO_2_, *Zea* was more efficient because it achieved high *A* despite lower *V*_pmax_ and *K*_CA_ by decreasing diffusional limitations and sustaining greater *C*_m_ with high *g*_m_. The high *g*_m_ at low *p*CO_2_ could increase or maintain photosynthesis at low *C*_i_ and could improve photosynthetic rates under situations that result in low CO_2_ availability, such as drought.

In both species, the conversion of CO_2_ into bicarbonate as catalysed by CA was fast enough that the hydration rate only limited *A* at low *p*CO_2_ (*L*_CA_=6–16% for *C*_m_<4 Pa, Fig. 4). Such low *C*_m_ is unlikely to occur, even under drought conditions. At these very low *p*CO_2_, the hydration rate (*V*_h_) was comparable to rates in CA-depleted transgenic plants (Supplementary Fig. S7). For example, in *Setaria* at 25 °C, *V*_h_ decreased from 581 μmol m^–2^ s^–1^ at ambient *p*CO_2_ to 100 μmol m^–2^ s^–1^ at the lowest *p*CO_2_ measured. Using values from Osborn *et al.* (2017) at 25 °C and ambient *p*CO_2_ to calculate *V*_h_ as *C*_m_×*K*_CA_ resulted in 1215 and 142 μmol m^–2^ s^–1^ for the wild type and CA-depleted transgenic, respectively. Osborn *et al.* (2017) concluded that in *Setaria* at low *p*CO_2_, *g*_m_ posed a greater limitation than CA activity. Our study confirms that *g*_m_ is a major determinant of photosynthetic capacity at low *p*CO_2_ and CA further constrains assimilation rates only at very low *p*CO_2_. However, the CA limitation at low *p*CO_2_ will be exacerbated at higher temperatures as the hydration rate is less able to keep up with the increase in PEPC activity (Boyd *et al.*, 2015).

### Leakiness (ϕ)

Leakiness is often estimated from comparing models and measurements of Δ^13^C assuming *g*_m_ is infinite (Pengelly *et al.*, 2010; Ubierna *et al.*, 2011, 2013) or large (Kromdijk *et al.*, 2010). Values of ϕ vary by as much as 0.04–0.9 (for a compilation of values and review of methods see Kromdijk *et al.*, 2014), although for most species under most conditions ϕ=0.2–0.3 (Cousins *et al.*, 2006; Kromdijk *et al.*, 2010; Pengelly *et al.*, 2010; Ubierna *et al.*, 2013; Bellasio and Griffiths, 2014).

In our study, considering *g*_m_ to be finite had a different effect on the calculation of ϕ for *Setaria* and *Zea*. At ambient air *p*CO_2_ and 25 °C, both *Setaria* and *Zea* had similar *g*_m_ (2.00 and 2.43 μmol m^–2^ s^–1^ Pa^–1^, respectively). However, while ϕ in *Zea* was the same whether *g*_m_ was finite or infinite, in *Setaria*, accounting for a finite *g*_m_ doubled ϕ (Fig. 5, compare Models 1 and 2). This high ϕ in *Setaria* was driven by constrains imposed by the Δ^13^C model rather than the photosynthesis model. This is illustrated by the comparison of Models 2 and 3 (Fig 5). Both models predicted the same *A* and ϕ, but Model 2 used *g*_m_ finite (and *in vitro V*_pmax_) and Model 3 assumed *g*_m_ infinite (and *in vivo V*_pmax_). However, Model 3 failed to predict $\Delta_{obs}^{13}$ (see Supplementary Fig. S2). Forcing the solution to satisfy both models of *A* and $\Delta_{obs}^{13}$ resulted in increases in ϕ in *Setaria*, but not in *Zea*.

**Fig. 6.**
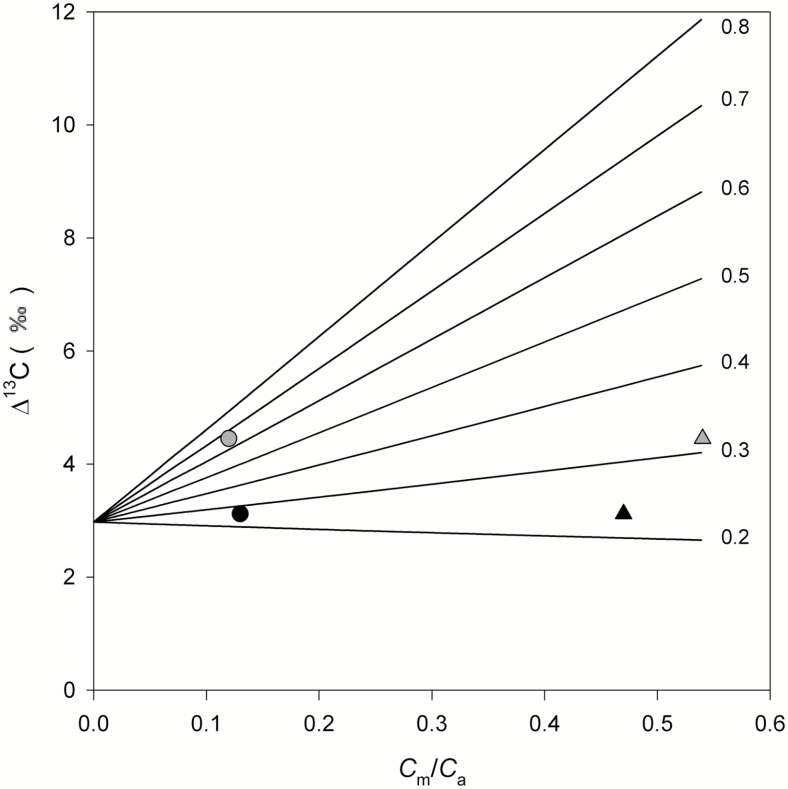
Δ^13^C (Eqn 9) as a function of *C*_m_/*C*_a_ for different ϕ values (indicated by the numbers at the end of each line). For calculations we used values of 37, 36, 20, and 1364 Pa for *C*_a_, *C*_L_, *C*_i_, and *C*_bs_, respectively; *t*=0.0058, *b*_4_=–4.49‰, and *b*_3_=29.87‰. These values correspond to the mean values measured or calculated in *Setaria* at 25 °C and ambient *p*CO_2_. Black symbols represent data for *Zea* and grey symbols for *Setaria*. For both species, ϕ was calculated assuming either *g*_m_ infinite (triangles) or *g*_m_=2.00 and 2.43 μmol m^–2^ s^–1^ Pa^–1^ in *Setaria* and *Zea*, respectively (circles).

This can be explained through the relationship between Δ^13^C and *C*_m_/*C*_a_, which is illustrated in Fig. 6 for different values of ϕ. Increasing *C*_m_/*C*_a_ results in either increased or decreased Δ^13^C depending on whether ϕ is low (≤0.3) or high (Henderson *et al.*, 1992; von Caemmerer *et al.*, 2014). When $\Delta_{obs}^{13}$ > *a*_s_ + (*a*_m_ – *a*_s_)(*C*_i_/*C*_a_) ( = 4.4–2.6 *C*_i_/*C*_a_ ≈ 2.9‰ in our data set at 25 ºC and ambient *p*CO_2_) increasing *C*_m_/*C*_a_ results in decreased ϕ; meanwhile the opposite is true when $\Delta_{obs}^{13}$ The value *a*_s_ + (*a*_m_ – *a*_s_)(*C*_i_/*C*_a_) represents the intercept of the line Δ^13^C versus *C*_m_/*C*_a_ when *C*_bs_ and boundary layer conductance are large and ternary effects are ignored. At ambient air *p*CO_2_ and 25 °C, $\Delta_{obs}^{13}$=3.1‰ in *Zea*. Therefore, varying *C*_m_/*C*_a_ resulted in minimal changes in ϕ (compare black triangle and circle in Fig. 6). However, in *Setaria*, $\Delta_{obs}^{13}$=4.5‰ and therefore low *C*_m_/Ca translated into large ϕ (compare grey triangle and circle in Fig. 6). The photosynthesis model demonstrated that this increase in ϕ was achieved by increased *V*_p_ and *g*_bs_ (see Supplementary Fig. S2).

It is questionable that *Setaria* operates with ϕ=0.7, and it is seemly unreasonable that it does. Because Δ^13^C is mostly determined by *C*_m_/*C*_a_ and ϕ, low *C*_m_/*C*_a_ forces the increase in ϕ. But are there any other parameters in the discrimination equation that could be manipulated in order to predict large Δ^13^C with low *C*_m_/*C*_a_ without large ϕ? Calculations of ϕ with the complete (Eqn 16) and simplified (Eqn 19) models suggest that, at least at ambient *p*CO_2_, this was not the case. The simplified calculation of ϕ produced values similar to the complete model, suggesting that at ambient air *p*CO_2_ or above, modifying parameters such as *C*_bs_, *b*_3_, or *b*_4_ within their current definition did not result in large changes in Δ^13^C.

In addition to the possible post-translational regulation of *V*_pmax_, PEP regeneration (*V*_pr_) may also influence *V*_p_ (Eqn 6) and estimates of ϕ. In our calculations, *V*_pr_=64 μmol m^–2^ s^–1^ decreased ϕ in *Setaria* by 0.3 and resulted in slightly larger *g*_m_ values at high *p*CO_2_ but no change at low *p*CO_2_ (compare Models 1 and 4 in Fig. 5 and Supplementary Fig. S2). In fact, at low *p*CO_2_ it is expected that *V*_pr_ would not limit *V*_p_ and would have no effect on estimates of *g*_m_ or ϕ under these conditions. Changes in ϕ in response to *p*CO_2_ or other conditions are possible if *V*_pr_ is allowed to vary, although at present *V*_pr_ variation across species, temperatures, or *p*CO_2_ is unknown. The *V*_pr_ values that would be needed to remove the observed trend in *g*_m_ with *p*CO_2_ are shown in Supplementary Fig. S8. Introducing a value for *V*_pr_ implies decoupling *V*_p_ from *C*_m_ (*g*_m_). In other words, the required *V*_p_ value to support the measured *A* could be achieved by choosing the adequate *V*_pr_ rather than by varying *C*_m_. This would also further complicate estimations of ϕ from Δ^13^C as *V*_pr_ is not often measured and is not incorporated into the Δ^13^C models.

Our calculations assume that theoretical models of photosynthesis and discrimination represent the actual photosynthetic process; any inaccuracy in the models will introduce error in the calculated *g*_m_. We have evaluated one common modelling simplification, the effect of CA limitation, and also the impact of uncertainty on input parameters. Additionally, we have used two contrasting species to illustrate the sensitivity of ϕ to *g*_m_. Although a complete analysis of ϕ is beyond the scope of this work, this should be undertaken in future studies together with investigations on PEP regeneration limitations. Other future foci for research include: investigating *in vivo* and *in vitro V*_pmax_ values and variation across species and environmental conditions; and compiling leaf structure, CA, aquaporins, or other data that could reveal potential mechanisms behind observed *g*_m_ patterns.

## Supplementary data

Supplementary data are available at *JXB* online.

Methods S1. Model of ^13^C discrimination in C_4_ species.

Table S1. Gas exchange values for *C*_i_ and *A*, and calculated values for *C*_m_ and CA-lim *g*_m_ in *Setaria viridis* and *Zea mays* at 25 °C and variable CO_2_ supply.

Fig. S1. *C*_m_ across *C*_i_ in *Setaria viridis* at three temperatures, and in *Zea mays* at 25 °C.

Fig. S2. Description of the models used to evaluate the effect of *g*_m_ in calculations of ϕ.

Fig. S3. Sensitivity of calculations of CA-lim *g*_m_ in *Setaria viridis* to uncertainty in input parameters.

Fig. S4. Impact of *R*_d_ and *b*′_3_ in the calculation of CA-lim *g*_m_ in *Setaria viridis* at 25 °C.

Fig. S5. Measured versus modeled response of *A* to *C*_i_ at 25 °C in *Setaria viridis* and *Zea mays* for different values of *V*_pmax_ and *g*_m_.

Fig. S6. Values for *in vivo V*_pmax_ across *C*_i_ in *Setaria viridis* calculated when CA-lim *g*_m_ is constant with *p*CO_2_.

Fig. S7. *V*_h_ across *C*_i_ in *Setaria viridis* at three temperatures.

Fig. S8. Values for *V*_pr_ across *C*_i_ in *Setaria viridis* calculated when CA-lim *g*_m_ is constant with *p*CO_2_.

Supplementary Figures

Supplementary Table 1

Supplementary Methods
