# Supplementary material for: The response of mesophyll conductance to short-term variation in CO2 in the C4 plants Setaria viridis and Zea mays
Source: J Exp Bot. 2018 Feb 21;69(5):1159–70. doi: 10.1093/jxb/erx464 (PMC6018935; doi:10.1093/jxb/erx464)
Supplement: Supplementary Figures [file erx464_suppl_supplementary_figures.pdf]

Supplementary Figure S1. Values for pCO_2 in the mesophyll (C_m, Pa) calculated with the carbonic anhydrase limited calculations across the pCO_2 inside the leaf intercellular spaces (C_i, Pa). Data are for *Setaria viridis* (black circles) at three temperatures (T_L = 40 ºC, panel A; T_L = 25 ºC, panel B; T_L = 10 ºC, panel C) and for *Zea mays* (grey squares) at T_L = 25 ºC. Values are means ± 1SE, n = 4 in *Setaria* and n = 3 in *Zea*. Arrows indicate values at ambient pCO_2.

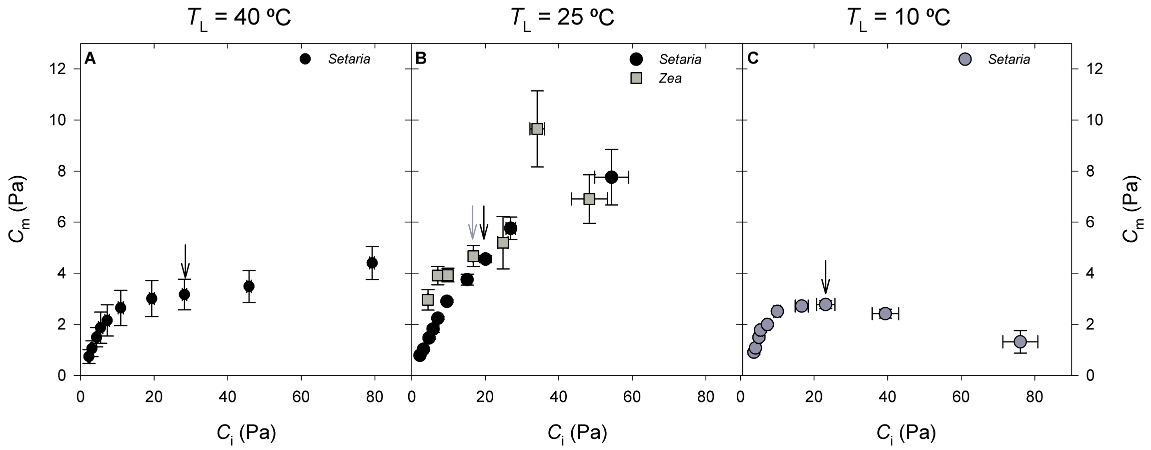

Supplementary Figure S2. Description of the models used to evaluate the effect of mesophyll conductance (g_m) in calculations of leakiness (ϕ). Model 1: uses *in -vitro* V_{pmax} and g_m finite and equal to the values presented in Fig. 3 of the main manuscript; Model 2: uses *in-vivo* V_{pmax} and g_m infinite; Model 3: is the same than Model 1 but the solution was only constrained by A and not $\Delta^{13}C$; Model 4: is the same than Model 1 but with $V_p = \min\left(V_{pr}, \frac{[HCO_3^-]V_{pmax}}{[HCO_3^-]+K_p}\right)$, where V_{pr} = 64 and 59 μmol $m^{-2}s^{-1}$ in *Setaria* and *Zea*, respectively. Several parameters that describe C_4 photosynthesis are plotted across the $p CO_2$ inside the leaf intercellular spaces (C_i, Pa), namely: maximal PEP carboxylation rate (V_{pmax}, μmol $m^{-2}s^{-1}$), mesophyll conductance (g_m, μmol $m^{-2}s^{-1}Pa^{-1}$), maximal Rubisco carboxylation rate (V_{cmax}, μmol $m^{-2}s^{-1}$), bundle-sheath conductance (g_{bs}, μmol $m^{-2}s^{-1}Pa^{-1}$), $p CO_2$ in the bundle-sheath cells (C_{bs}, Pa), PEP carboxylation rate (V_p, μmol $m^{-2}s^{-1}$) and Rubisco carboxylation rate (V_c, μmol $m^{-2}s^{-1}$). All models applied the CA-limited calculations to *Setaria virids* and *Zea mays* data measured at 25 ºC.

The models of A (photosynthetic rate, μmol $m^{-2}s^{-1}$) and $\Delta^{13}C$ (^{13}C discrimination, ‰) include four variables − C_m, V_{pmax}, g_{bs} and V_{cmax}− that are grouped in the pairs (C_m, V_{pmax}) and (g_{bs}, V_{cmax}), with the exception of the crossed term $g_{bs}C_m$ that is very small. This explains why one pair can be calculated as a function of the other pair rather than as a function of the individual values for each variable (detailed explanation can be found in Ubierna *et al.*, 2017). To illustrate that individual values chosen for V_{cmax} and g_{bs} do not affect the calculation of g_m or ϕ, the four models were applied in two set of conditions: considering V_{cmax} either variable (**A**) or constant (**B**) with $p CO_2$. In **A** we used *in-vivo* V_{cmax}, and in **B** we used V_{cmax} = 34 and 31 μmol $m^{-2}s^{-1}$ for *Setaria* and *Zea*, respectively. Therefore, Figure S2 includes the following sub-figures:

Fig. S2.A.1. Models 1 to 4 in *Setaria virids* when V_{cmax} is variable with $p CO_2$
Fig. S2.A.2. Models 1 to 4 in *Zea mays* when V_{cmax} is variable with $p CO_2$
Fig. S2.B.1. Models 1 to 4 in *Setaria virids* when V_{cmax} is constant with $p CO_2$
Fig. S2.B.2. Models 1 to 4 in *Zea mays* when V_{cmax} is constant with $p CO_2$

Figure S2.A.1 Models 1 to 4 in *Setaria virids* when V_{cmax} is variable with pCO_2

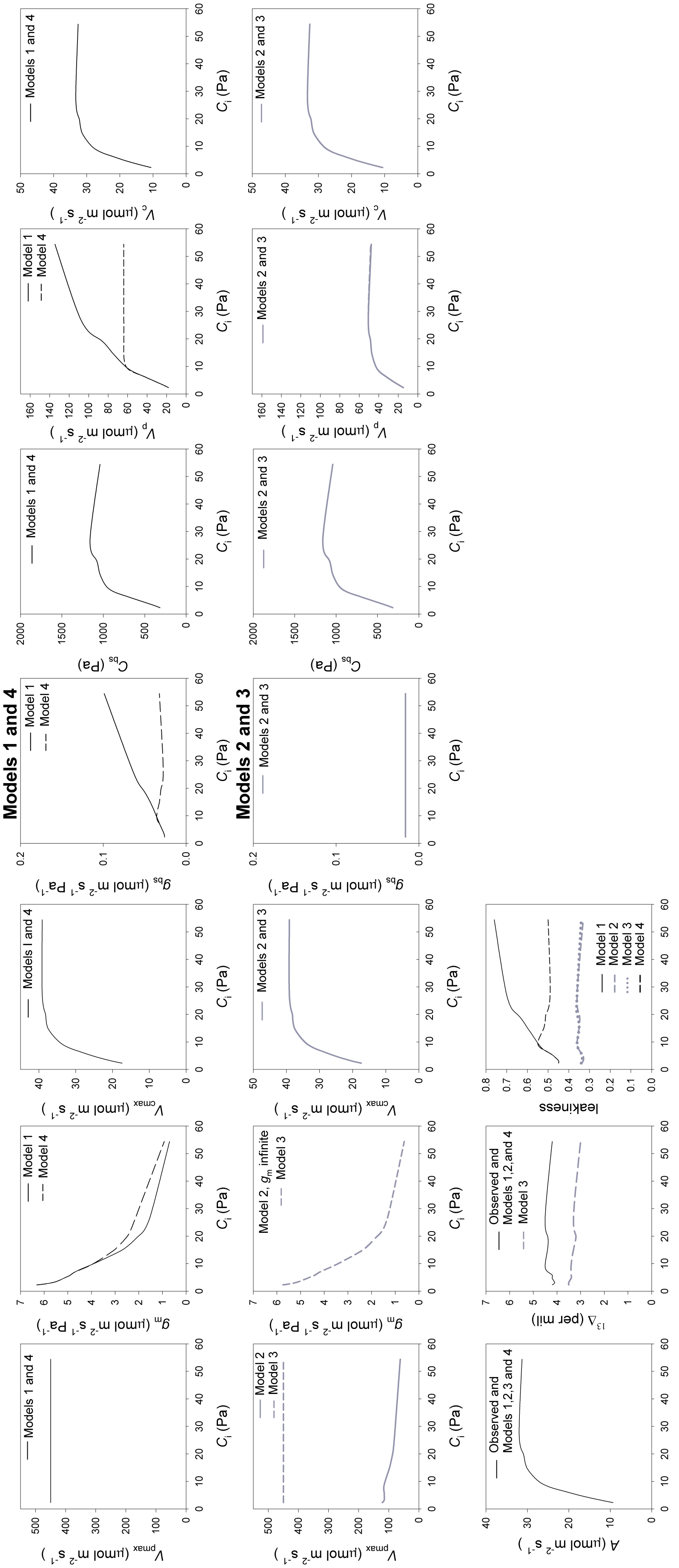

Figure S2.A.2 Models 1 to 4 in *Zea mays* when V_{cmax} is variable with pCO_2

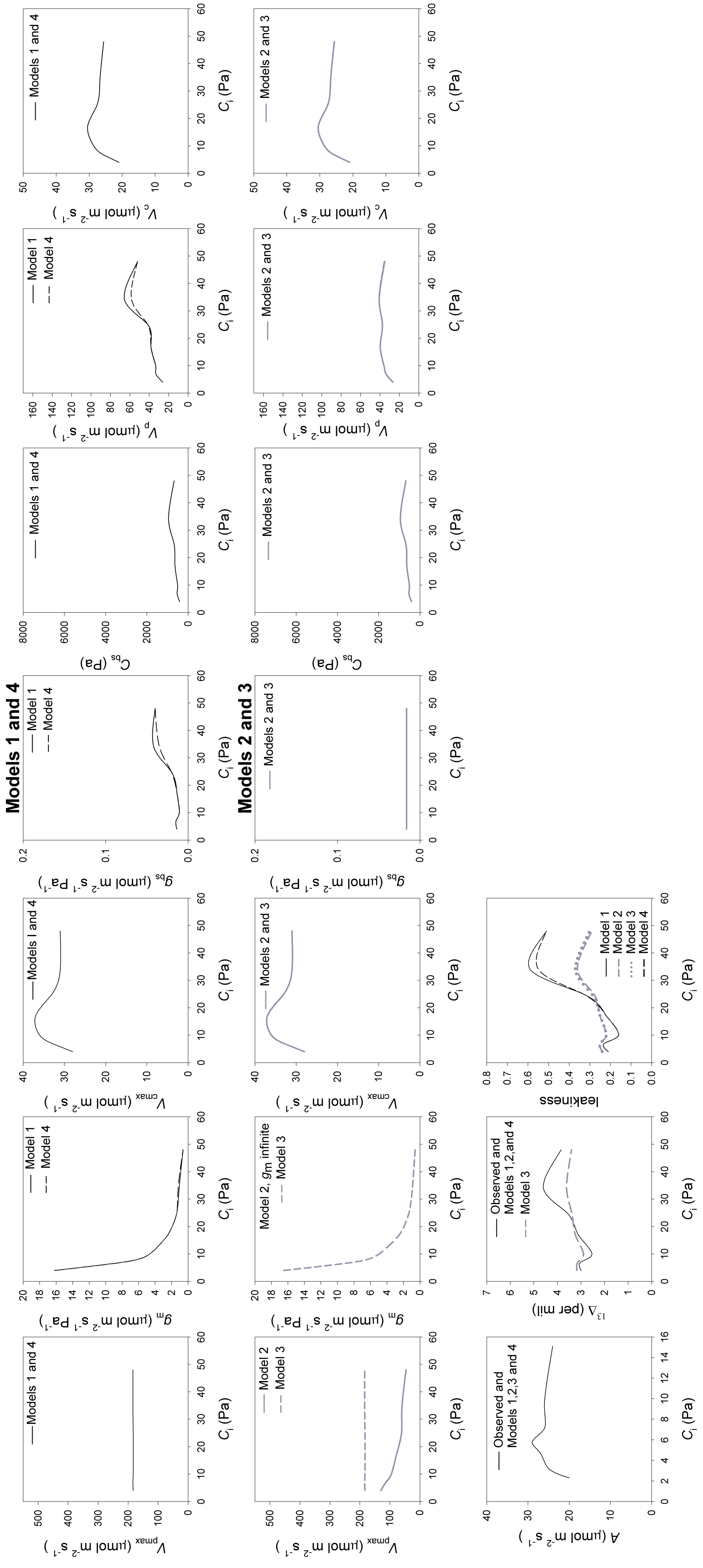

Figure S2.B.1 Models 1 to 4 in *Setaria virids* when V_{cmax} is constant with pCO_2

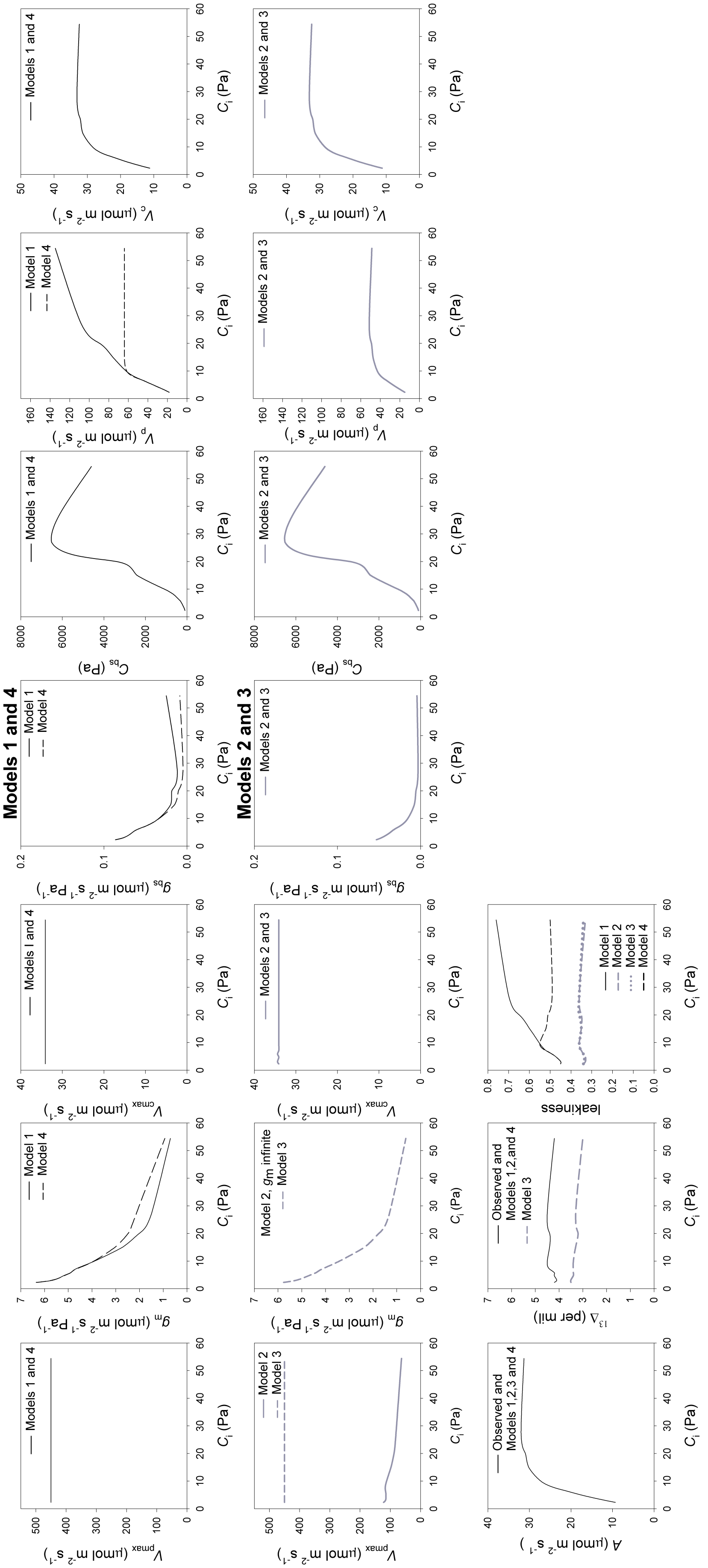

Figure S2.B.2 Models 1 to 4 in *Zea mays* when V_{cmax} is constant with pCO_2

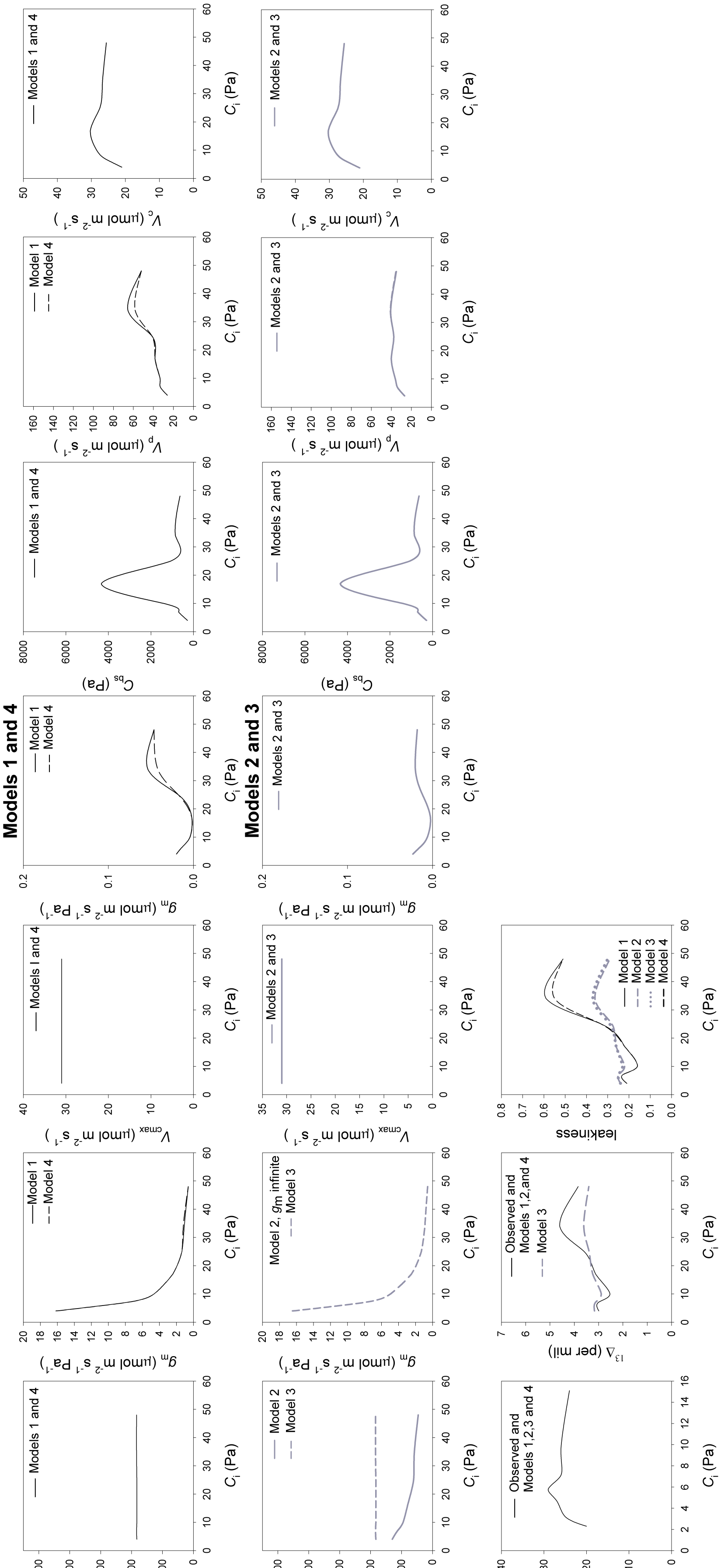

Supplementary Figure S3. Sensitivity of calculations of carbonic anhydrase limited mesophyll conductance (CA-lim g_m) in *Setaria viridis* to uncertainty in values for Michaelis-Menten constant of PEPC (K_P, **A - C**), rate constant of CA for CO_2 (K_{CA}, **D - F**), Michaelis-Menten constant of Rubisco (K_C, **G- I**), and maximal PEP carboxylation rate (V_{pmax}, **J - L**). Calculations were performed at three temperatures (10, 25 and 40 ºC) and three pCO_2 (C_a = 93, 38 and 5-10 Pa for high - solid line -, ambient - dashed line -, and low - dashed and dotted line -, respectively). The numbers on top of each panel represent the actual parameter value at each temperature. Each parameter was varied ± 20 and ± 50% of its actual value and the % change in g_m was calculated by comparing CA-lim g_m results calculated with the assumed versus the actual parameter value.

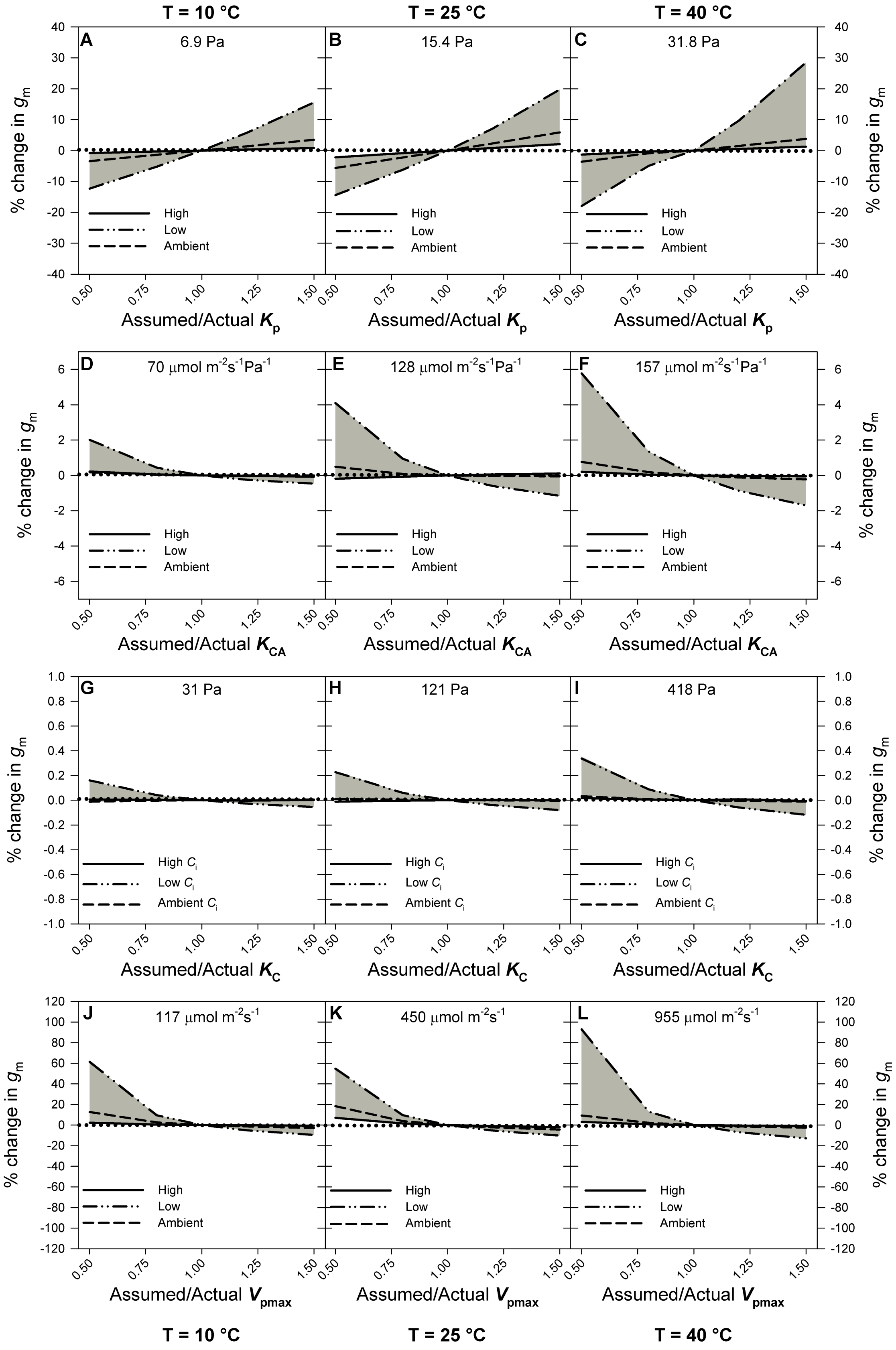
T = 10 °C
T = 25 °C
T = 40 °C
A 6.9 Pa
B 15.4 Pa
C 31.8 Pa
% change in g_m
High
Low
Ambient
Assumed/Actual K_p
D 70 μmol m^{-2}s^{-1}Pa^{-1}
E 128 μmol m^{-2}s^{-1}Pa^{-1}
F 157 μmol m^{-2}s^{-1}Pa^{-1}
Assumed/Actual K_{CA}
G 31 Pa
H 121 Pa
I 418 Pa
High C_i
Low C_i
Ambient C_i
Assumed/Actual K_C
J 117 μmol m^{-2}s^{-1}
K 450 μmol m^{-2}s^{-1}
L 955 μmol m^{-2}s^{-1}
Assumed/Actual V_{pmax}
T = 10 °C
T = 25 °C
T = 40 °C

Supplementary Figure S4. Impact of R_d (non-photorespiratory CO_2 released in the dark) and b_3' (Rubisco fractionation) in the calculation of carbonic anhydrase limited mesophyll conductance (CA-lim g_m) in *Setaria viridis* at 25 ºC. **A**) and **C**) means ± 1SE, n =4. **B**) and **D**) % change in CA-lim g_m. The reference CA-lim g_m values for calculating the percentages were CA-lim g_m calculated with measured R_d in **B** and CA-lim g_m calculated with b_3' = 30‰ in **D**. Arrows indicate values at ambient pCO_2.

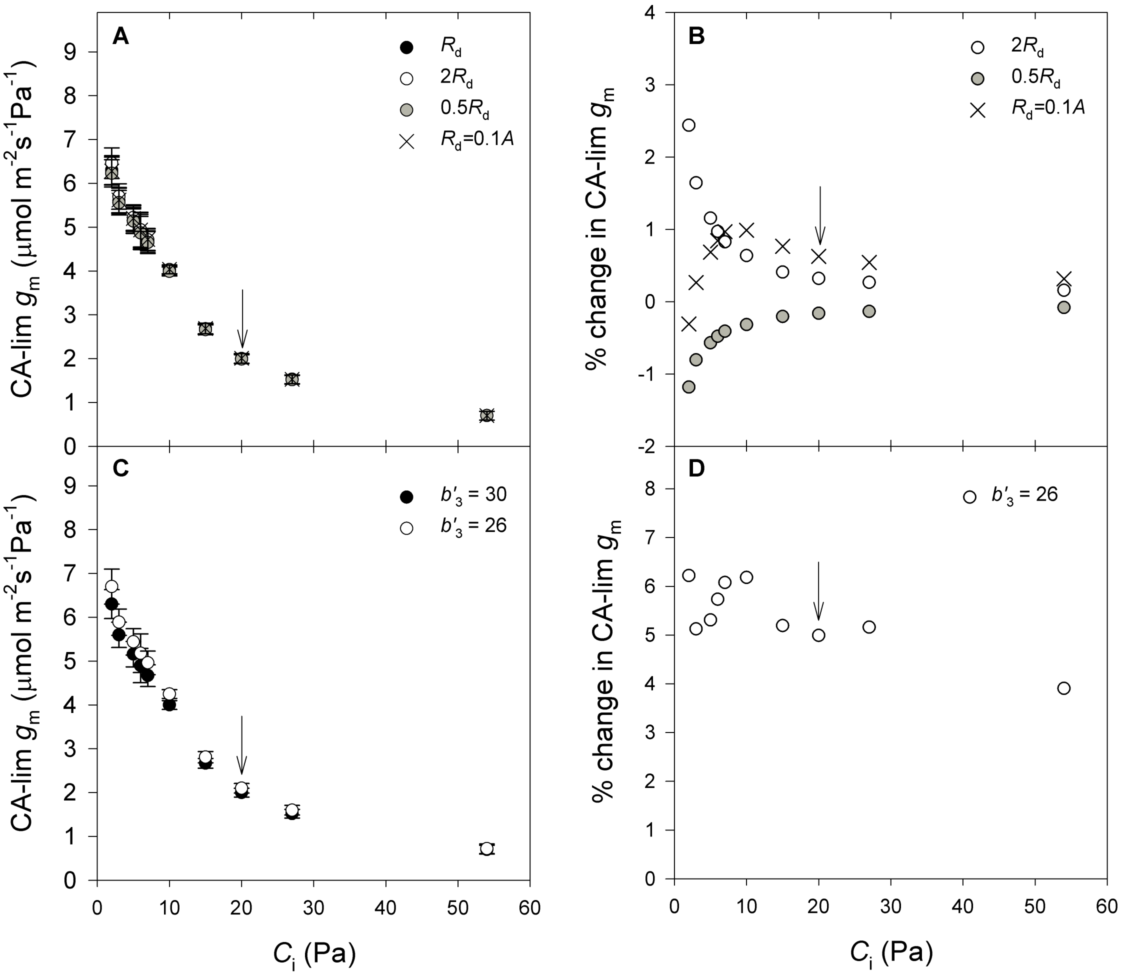

Supplementary Figure S5. Measured (symbols) versus modeled (black lines) response of photosynthetic rate (A) to variation in the leaf intercellular CO_2 partial pressure (C_i) at 25 ºC in *Setaria viridis* (**A**, **C**) and *Zea mays* (**B**, **D**). In *Zea*, the data are for a different set of 4 plants than the set used in the main document which includes more data points at low pCO_2. In all panels modeled A was calculated with Eqn 1, assuming CA-limiting and $V_{cmax} \approx A_{max} + R_d$. In panels **A** and **B** modeled A was calculated assuming $V_{pmax} = 450$ (*Setaria*) or 184 µmol m^{-2} s^{-1} (*Zea*) and C_m equal to C_i (solid line), 0.5C_i (dashed-dotted), 0.25C_i (dashed) or 0.15C_i (dotted). In panels **C** and **D** modeled A was calculated assuming $C_m = C_i$ and V_{pmax} equal to 450 µmol m^{-2} s^{-1} (solid line), 225 µmol m^{-2} s^{-1} (dashed-dotted), 184 µmol m^{-2} s^{-1} (dashed) or 92 µmol m^{-2} s^{-1} (dotted). Values are means ± 1SE, $n = 4$. The arrows indicate the values at ambient pCO_2. The grey lines in panels **A** and **B** are the function fitted to the means $A = a(1 - e^{-bC_i}) + c$. Function fitting was done with SigmaPlot 11.0 (Systat Software Inc., San Jose, California, USA). *Setaria*: a = 35.64 ± 1.18, b = 0.19 ± 0.01, c = -3.72 ± 1.26, r^2 = 1.00, $F(2,7)$ = 1059, P < 0.0001. *Zea*: a = 30.59 ± 0.52, b = 0.26 ± 0.01, c = 1.22 ± 0.51, r^2 = 1.00, $F(2,9)$ = 2280, P < 0.0001.

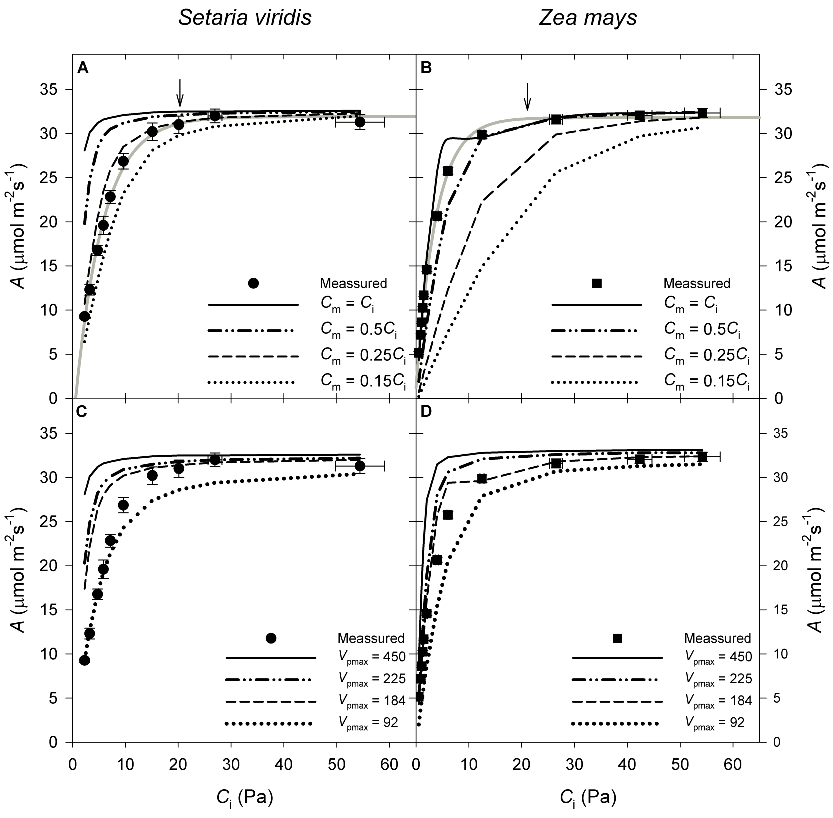

Supplementary Figure S6. Variation in maximum PEP carboxylation rate (V_{pmax}, µmol m^{-2}s^{-1}) across pCO_2 that is required to match observations of A and $\Delta^{13}C$ in *Setaria viridis* with their theoretical predictions assuming that mesophyll conductance (CA-lim g_m) is constant with pCO_2 and equal to 2.7 ± 0.3, 6.3 ± 0.3 and 9.5 ± 1.4 µmol m^{-2} s^{-1} Pa^{-1} at 10, 25, and 40 ºC, respectively. These values correspond to the maximum CA-lim g_m values at each temperature derived for *Setaria viridis* with the *in-vitro* V_{pmax} method. Values are means ± 1SE, n = 4.

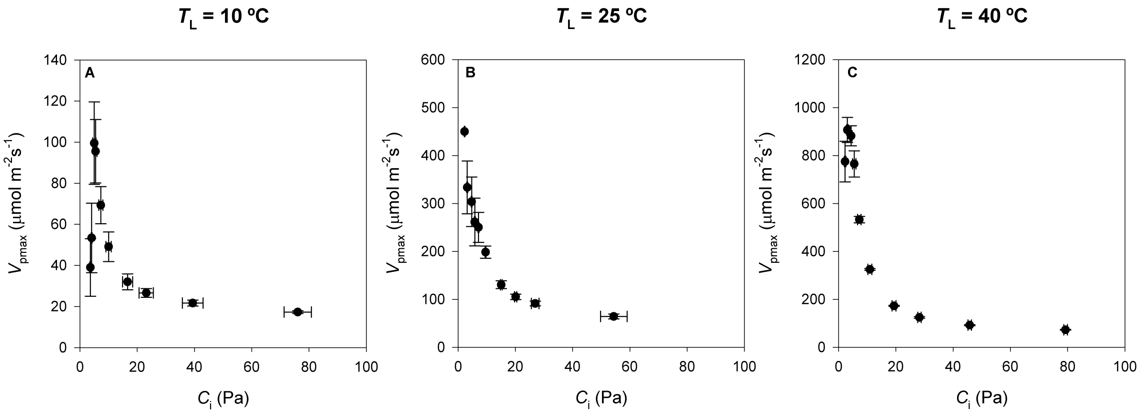

Supplementary Figure S7. Hydration rate ($V_h = C_m * K_{CA}$) in function of pCO_2 inside the leaf intercellular spaces (C_i) in *Setaria viridis* (**A**) and *Zea mays* (**B**). Values for three leaf temperatures (T_L) are presented in *Setaria*: 10 ºC (grey circles), 25 ºC (black circles) and 40 ºC (white circles), and for T_L = 25 ºC in *Zea*. Values are means ± 1 SE, n = 4 (*Setaria*) or 3 (*Zea*). The values for the rate constant of carbonic anhydrase (CA) for CO_2 at 25ºC are: K_{CA} = 65.5 µmol m^{-2} s^{-1} Pa^{-1} (*Zea*) and K_{CA} = 124 µmol m^{-2} s^{-1} Pa^{-1} (*Setaria*) (R.A. Boyd unpublished, Boyd *et al.*, 2015) and varying with temperature as described in Boyd *et al.* (2015).

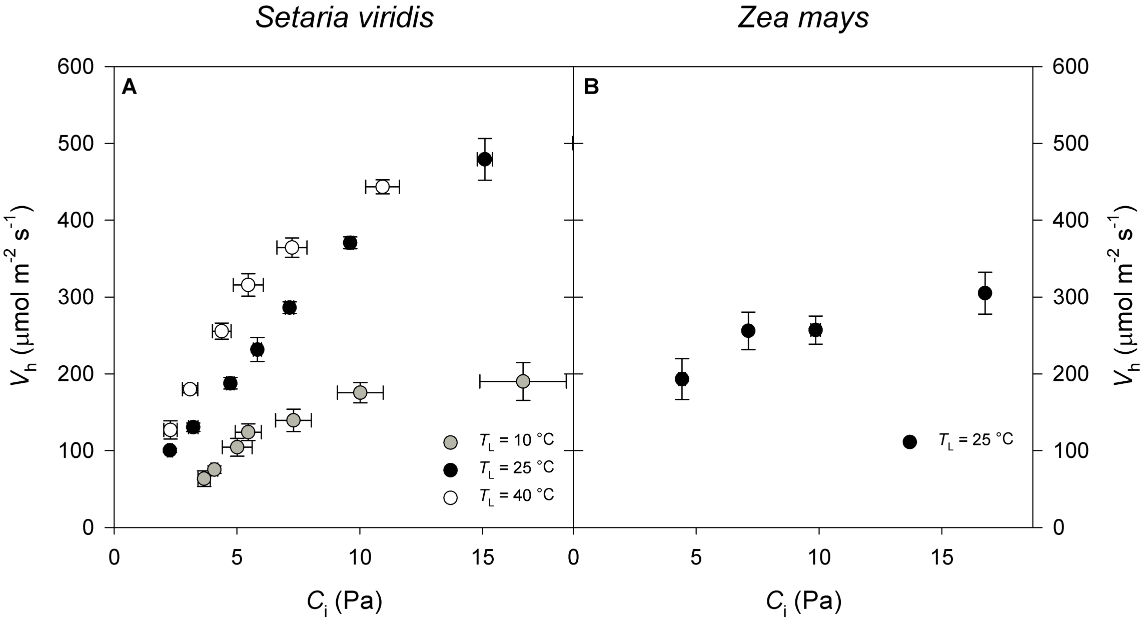

Supplementary Figure S8. PEP regeneration rate (V_{pr}) as a function of pCO$_2$ inside the leaf intercellular spaces (C_i) needed to match observations of A and Δ^{13}C in *Setaria viridis* with their theoretical predictions assuming that mesophyll conductance (CA-lim g_m) is constant with pCO$_2$ and equal to 2.7 ± 0.3, 6.3 ± 0.3 and 9.5 ± 1.4 µmol m^{-2} s^{-1} Pa^{-1} at 10, 25, and 40 ºC, respectively. These values correspond to the maximum CA-lim g_m values derived in *Setaria viridis* at each temperature with the *in-vitro* V_{pmax} method. Values are means ± 1SE, n = 4.

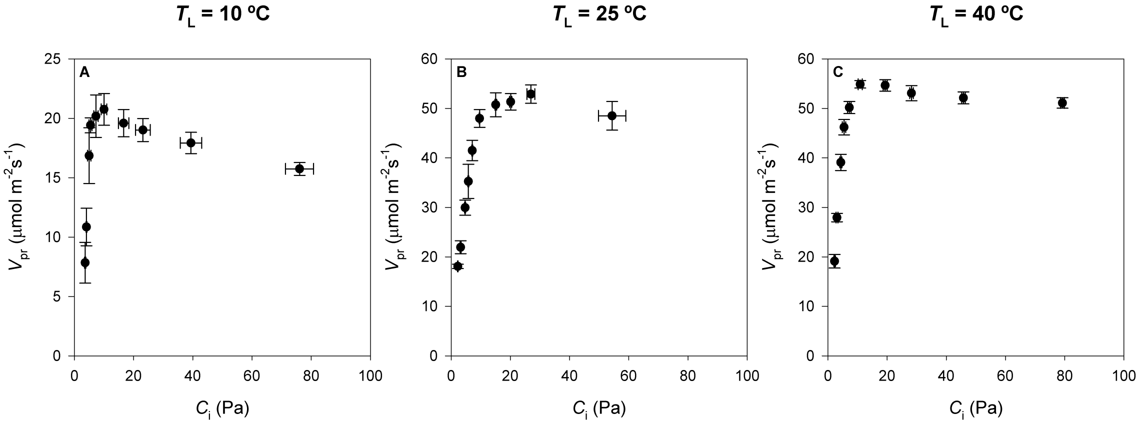

References

von Caemmerer S. 2000. *Biochemical models of leaf photosynthesis*. Collingwood, Australia: CSIRO publishing.

Boyd RA, Gandin A, Cousins AB. 2015. Temperature responses of C$_4$ photosynthesis: biochemical analysis of Rubisco, Phospho*enol*pyruvate carboxylase, and Carbonic anhydrase in *Setaria viridis*. *Plant Physiology* **169**, 1850–1861.

Ubierna N, Gandin A, Boyd RA, Cousins AB. 2017. Temperature response of mesophyll conductance in three C$_4$ species calculated with two methods: ^{18}O discrimination and *in-vitro* V_{pmax}. *New Phytologist* **214**, 66-80.
