# Supplementary material for: The response of mesophyll conductance to short-term variation in CO2 in the C4 plants Setaria viridis and Zea mays
Source: J Exp Bot. 2018 Feb 21;69(5):1159–70. doi: 10.1093/jxb/erx464 (PMC6018935; doi:10.1093/jxb/erx464)
Supplement: Supplementary Table 1 [file erx464_suppl_supplementary_table_1.pdf]

Supplementary Table S1. The CO_2 partial pressure drop between the ambient air (C_a) and the leaf intercellular spaces (C_i) and between C_i and the mesophyll (C_m) expressed as a proportion of the total gradient C_a - C_m in *Setaria viridis* and *Zea mays* measured at 25 ºC. The C_m was derived with the CA-limited calculations. Values for CO_2 assimilation rate (A) and CA-lim g_m are also presented. Values are means ± 1SE, n = 4 in *Setaria* and n = 3 in *Zea*.

C_a Pa	C_i Pa	C_m Pa	C_a-C_i %	C_i-C_m %	A µmol m^{-2} s^{-1}	g_m µmol m^{-2} s^{-1} Pa^{-1}
Setaria viridis T_L = 25 ºC						
4.7 ± 0.0	2.3 ± 0.1	0.8 ± 0.0	62	38	9.3 ± 0.3	6.30 ± 0.32
6.5 ± 0.1	3.2 ± 0.2	1.0 ± 0.0	59	41	12.3 ± 0.6	5.60 ± 0.29
9.3 ± 0.0	4.7 ± 0.2	1.5 ± 0.1	58	42	16.8 ± 0.6	5.16 ± 0.29
11.2 ± 0.1	5.8 ± 0.2	1.8 ± 0.1	57	43	19.6 ± 1.0	4.90 ± 0.39
13.9 ± 0.0	7.1 ± 0.2	2.2 ± 0.1	58	42	22.8 ± 0.7	4.67 ± 0.25
18.5 ± 0.1	9.6 ± 0.1	2.9 ± 0.1	57	43	26.9 ± 0.9	4.00 ± 0.10
27.9 ± 0.1	15.1 ± 0.3	3.8 ± 0.2	53	47	30.2 ± 1.0	2.67 ± 0.11
37.3 ± 0.1	20.1 ± 0.4	4.6 ± 0.1	53	47	31.0 ± 1.0	2.00 ± 0.10
56.0 ± 0.2	27.0 ± 1.3	5.8 ± 0.4	58	42	32.0 ± 0.8	1.52 ± 0.10
92.8 ± 0.2	54.4 ± 4.6	7.8 ± 1.1	45	55	31.3 ± 0.9	0.70 ± 0.10
Zea mays T_L = 25 ºC						
9.4 ± 0.0	4.4 ± 0.1	3.0 ± 0.4	77	23	19.7 ± 1.0	16.20 ± 5.74
13.9 ± 0.0	7.1 ± 0.0	3.9 ± 0.4	68	32	24.9 ± 1.0	7.92 ± 1.18
18.6 ± 0.1	9.9 ± 0.2	3.9 ± 0.3	60	40	27.4 ± 0.5	4.64 ± 0.31
35.5 ± 0.1	16.8 ± 0.1	4.7 ± 0.4	61	39	29.3 ± 0.4	2.43 ± 0.13
56.0 ± 0.3	24.8 ± 1.4	5.2 ± 1.0	61	39	26.4 ± 0.8	1.36 ± 0.12
84.1 ± 0.4	34.1 ± 2.0	9.6 ± 1.5	67	33	25.6 ± 0.9	1.07 ± 0.13
112.1 ± 0.6	48.3 ± 4.9	6.9 ± 1.0	61	39	24.4 ± 0.0	0.60 ± 0.07
