# Supplementary material for: The response of mesophyll conductance to short-term variation in CO2 in the C4 plants Setaria viridis and Zea mays
Source: J Exp Bot. 2018 Feb 21;69(5):1159–70. doi: 10.1093/jxb/erx464 (PMC6018935; doi:10.1093/jxb/erx464)
Supplement: Supplementary Methods [file erx464_suppl_supplementary_methods.pdf]

Supplementary Methods S1: model of ^{13}C discrimination in C_4 species

The *theo*retical model for photosynthetic discrimination against ^{13}C is (Farquhar and Cernusak, 2012):

$$\Delta^{13}_{\text{theo}} = \frac{1}{1-t}\left[a_{\text{b}}\frac{C_{\text{a}}-C_{\text{L}}}{C_{\text{a}}} + a_{\text{s}}\frac{C_{\text{L}}-C_{\text{i}}}{C_{\text{a}}}\right] + \frac{1+t}{1-t}\left[a_{\text{m}}\frac{C_{\text{i}}-C_{\text{m}}}{C_{\text{a}}} + \frac{b_4+\phi\left(\frac{b_3 C_{\text{bs}}}{C_{\text{bs}}-C_{\text{m}}} - s\right)}{1+\frac{\phi C_{\text{m}}}{C_{\text{bs}}-C_{\text{m}}}}\frac{C_{\text{m}}}{C_{\text{a}}}\right] \quad \text{Eqn S1.1}$$

where C_{a}, C_{L} $\left(C_{\text{L}} = C_{\text{a}} - \frac{1.37A}{g_{\text{bl}}}\right.$, with g_{bl} = boundary layer conductance$\left.\right)$, C_{i}, and C_{bs} $\left(C_{\text{bs}} = \frac{\gamma^* O_s + K_c\left(1+\frac{O_s}{K_O}\right)\left(\frac{A+R_d}{V_{cmax}}\right)}{1-\frac{A+R_d}{V_{cmax}}}\right.$, von Caemmerer 2000$\left.\right)$ are the pCO$_2$ in the chamber air, at the leaf surface, inside the leaf, and in the bundle-sheath cells respectively. The t $\left(t = \frac{\alpha_{ac}E}{2g_{ac}}\right.$, Farquhar & Cernusak, 2012$\left.\right)$ is the ternary correction coefficient where E is measured transpiration rate, g_{ac} is the conductance to diffusion of CO_2 in air and $\alpha_{ac} = 1 + \bar{a}$, where $\bar{a}$ $\left(= \frac{a_b(C_a-C_L)+a_s(C_L-C_i)}{C_a-C_i}\right)$ is the fractionation for combined ^{13}C diffusion across the stomata and boundary layer. The a_{b}, a_{s}, a_{m}, and s are the ^{13}C fractionation during diffusion through the leaf boundary layer (2.9‰), diffusion in air (4.4‰), liquid phase diffusion and dissolution (1.8‰), and leakage of CO_2 out of the bundle-sheath cells (1.8‰), respectively. The b_3 (^{13}C fractionation during carboxylation by Rubisco) and b_4 (net fractionation by CO_2 dissolution, hydration and PEPc) are calculated as (Farquhar, 1983):

$$b_3 \cong b_3' - \frac{eR_d}{V_c} - \frac{0.5fV_o}{V_c} \quad \text{Eqn S1.2}$$

$$b_4 = b_4'\left(1-\frac{V_p}{V_h}\right) + (e_s + h)\frac{V_p}{V_h} - \frac{eR_m}{V_p} \quad \text{Eqn S1.3}$$

where b_3'= 30‰; $b_4' = \left(\frac{-9.483*1000}{273+T} + 23.89 + 2.2\right)$‰ ($T$ is leaf temperature in Celsius); f, h, e_{s} and e, are the ^{13}C fractionations during photorespiration (11.6‰, Lanigan *et al.*, 2008), catalysed CO_2 hydration (1.1‰, Cousins *et al.*, 2006), CO_2 dissolution (1.1‰, O´Leary, 1984), and decarboxylation, respectively. The $e = e_{\text{Rd}} + e^*$, where $e^* = \delta^{13}_{\text{a}}$ - Δ_{obs} - $\delta^{13}_{substrate}$ (Wingate *et al.*, 2007; Cernusak *et al.*, 2013). We used e_{Rd} = 0 and $e^* \approx \delta^{13}_{\text{a}}$ – (-8), which resulted in average values of 2.0 and 1.9‰ for *Setaria* and *Zea*, respectively. Rubisco carboxylation (V_{c}), and Rubisco oxygenation (V_{o}) rates are calculated as V_{c}

$= \frac{C_{bs}V_{cmax}}{C_{bs}+K_C\left(1+\frac{O_S}{K_O}\right)}$, and $V_o = V_c\frac{2\Gamma^*}{C_{bs}}$, respectively, with $\Gamma^* = O_S\gamma^*$ (von Caemmerer, 2000).

Hydration rate (V_h) is C_m*K_{CA} where K_{CA} is the rate constant of CA for CO_2 and PEP carboxylation rate (V_p) is $V_p = \left\{\begin{matrix} \text{CA saturated} \rightarrow \frac{C_m V_{pmax}}{C_m + K_p} \\ \text{CA limited} \rightarrow \frac{[HCO_3^-]V_{pmax}}{[HCO_3^-]+K_p} \end{matrix}\right\}$.

References

von Caemmerer S. 2000. *Biochemical models of leaf photosynthesis*. Collingwood, Australia: CSIRO publishing.

Cernusak LA, Ubierna N, Winter K, Holtum JAM, Marshall JD, Farquhar GD. 2013. Environmental and physiological determinants of carbon isotope discrimination in terrestrial plants. *New Phytologist* **200**, 950–965.

Cousins AB, Badger MR, von Caemmerer S. 2006. Carbonic anhydrase and its influence on carbon isotope discrimination during C_4 photosynthesis. Insights from antisense RNA in *Flaveria bidentis*. *Plant Physiology* **141**, 232-242.

Farquhar GD. 1983. On the nature of carbon isotope discrimination in C_4 species. *Australian Journal of Plant Physiology* **10**, 205-226.

Farquhar GD, Cernusak LA. 2012. Ternary effects on the gas exchange of isotopologues of carbon dioxide. *Plant, Cell & Environment* **35**, 1221-1231.

Lanigan GJ, Betson N, Griffiths H, Seibt U. 2008. Carbon isotope fractionation during photorespiration and carboxylation in *Senecio*. *Plant Physiology* **148**, 2013-2020.

O'Leary MH. 1984. Measurement of the isotope fractionation associated with diffusion of carbon dioxide in aqueous solution. *Journal of Physical Chemistry* **88**, 823-825.

Wingate L, Seibt U, Moncrieff JB, Jarvis PG, Lloyd J. 2007. Variations in ^{13}C discrimination during CO_2 exchange by *Picea sitchensis* branches in the field. *Plant, Cell & Environment* **30**, 600-616.
